# Watershed, climate, and stable isotope data (oxygen-18 and deuterium) for 50 boreal lakes in the oil sands region, northeastern Alberta, Canada, 2002–2017

**DOI:** 10.1016/j.dib.2020.105308

**Published:** 2020-02-24

**Authors:** J.J. Gibson, Y. Yi, S.J. Birks

**Affiliations:** a InnoTech Alberta, 3-4476 Markham Street, Victoria, BC, V8Z 7X8, Canada; b University of Victoria, Department of Geography, Victoria, BC, V8W 3R4, Canada; c Environmental Monitoring and Science Division, Alberta Environment and Parks, Edmonton, T5J 5C6, Canada; d InnoTech Alberta, 3608 - 33 St NW, Calgary, AB, T2L 2A6, Canada

**Keywords:** Stable isotopes, Lakes, Water balance, Boreal wetlands, Oil sands environment, Permafrost thaw

## Abstract

Watershed data, climate and stable data collected over a 16-year period from a network of 50 lakes in northeastern Alberta, are provided to allow for broader incorporation into regional assessments of environmental impacts, particularly hydrologic and geochemical processes under changing climate and land use development. Oxygen-18 and deuterium analyses of water samples are provided from late summer surveys of 50 lakes with varying land cover and permafrost conditions. Six sub-groups of lakes are represented, including Stony Mountains, West Fort McMurray, Northeast Fort McMurray, Birch Mountains, Caribou Mountains and Shield. This dataset includes 1582 isotopic analyses made on 791 water samples and 3164 isotope mass balance model outputs, as well as 800 lake/watershed parameters, 5600 climate parameters, and 800 modelled values for isotopic composition of precipitation used in the computations. Model data are provided to facilitate evaluation of transferability of the model for other applications, and to permit more sophisticated spatial analysis and intercomparison with geochemical and biological datasets. Details and further discussion on the isotope mass balance approach are provided in “Regional trends in water balance and runoff to fifty boreal lakes: a 16-year isotope mass balance assessment including evaluation of hydrologic drivers” [1]. Overall, the data are expected to be useful, in comparison with local and regional datasets, for water resource management and planning, including design of monitoring networks and environmental impact assessments for oil sands projects.

**Table:** Specifications Table

Subject area	Water resources, hydrology
More specific subject area	Lake and watershed hydrology
Type of data	Tables, figure,.xlsx file
How data were acquired	Lake and watershed data are based on field measurements as well as digital elevation model data, hydrographic network data and maps; wetland classifications are based on 1:20:000 vertical air photo interpretation; drift thickness and distance to buried channels are based on geologic and hydrostratigraphic data available online from Alberta Geological Survey; climate data are interpolated from the North American Regional Reanalysis (NARR) monthly climatology; monthly δ^18^O and δ^2^H in precipitation were amount-weighted using NARR monthly precipitation obtained from the NARR dataset. Isotope balance is based on a commonly-applied model using evaporation-flux-weighted δ^18^O and δ^2^H in atmospheric moisture, relative humidity and precipitation. Lake depth and volume were based on on-site bathymetry. ArcGIS/ArcHYDRO was used for spatial analysis; GrADs was used for spatial interpolation.
Data format	Raw isotope analytical data are reported in per mil relative to Vienna Standard Mean Ocean Water (‰ VSMOW) and normalized to SMOW/SLAP (Standard Light Antarctic Precipitation); raw climate data, lake data, watershed data, and land cover data are reported for each site. Evaporation/inflow, water yield/precipitation are reported as percentages (ratios X 100%); water yield to lakes is reported in millimetres per year (mm/year), residence times of lakes is reported in years. Mann-Kendall statistics, including tau and p-values are provided for all sites/years based on the R code (https://www.R-project.org/).
Experimental factors	Water samples were collected by float plane or helicopter in 30-mL high density polyethylene (HDPE) bottles ensuring lids were tightly sealed and stored at room temperature prior to analysis; Spatially representative climate data (temperature, relative humidity, precipitation and evaporation) were obtained from North American Regional Reanalysis (NARR); lake and watershed areas were delineated from a 30-m digital elevation model.
Experimental features	The lakes, deemed to be acid sensitive, were selected by the Regional Aquatic Monitoring Program (RAMP) and are situated in remote locations without road access, and during 2002–2017, have generally been sampled annually in August/September.
Data source location	Northeastern Alberta, Canada between 55.68°N and 59.72°N, and between 110.02°W and 115.46°W. (http://www.ramp-alberta.org/RAMP.aspx).
Data accessibility	Watershed, climate, and stable isotope data are stored within this article. Geochemical data are available from the RAMP Program (http://www.ramp-alberta.org/data/AcidSensitiveLakes/default.aspx).
Related research article	Gibson, J.J., Yi, Y., Birks, S.J., Isotopic tracing of hydrologic drivers including permafrost thaw status for lakes across northeastern Alberta, Canada: a 16-year, 50-lake perspective. Journal of Hydrology Regional Studies 26, 100,643. https://doi.org/10.1016/j.ejrh.2019.100643 [1].

**Table:** 

**Value of the data** • Interannual time-series dataset over a 16-year period at 50 sites offering new insight into isotopic labelling of water cycle components, useful for assessment of evaporation losses, water yield, residence time of lakes, climate change and critical loads assessment. • Values and trends in hydrologic indicators are expected to be useful for understanding significant climate, water balance and geochemical changes occurring at the sites, including significant pH increases in lakewater. • Statistical analysis of spatial and temporal trends in raw data and model outputs may be informative for evaluation of climate and environmental changes across the region, and area under significant development pressure owing to oil sands mining and insitu production. Isotopic and model data may also be useful for designing regional monitoring programs, to ensure that the full range of water budget conditions and controlling factors are considered

## 1 Data

**Fig. 1:**
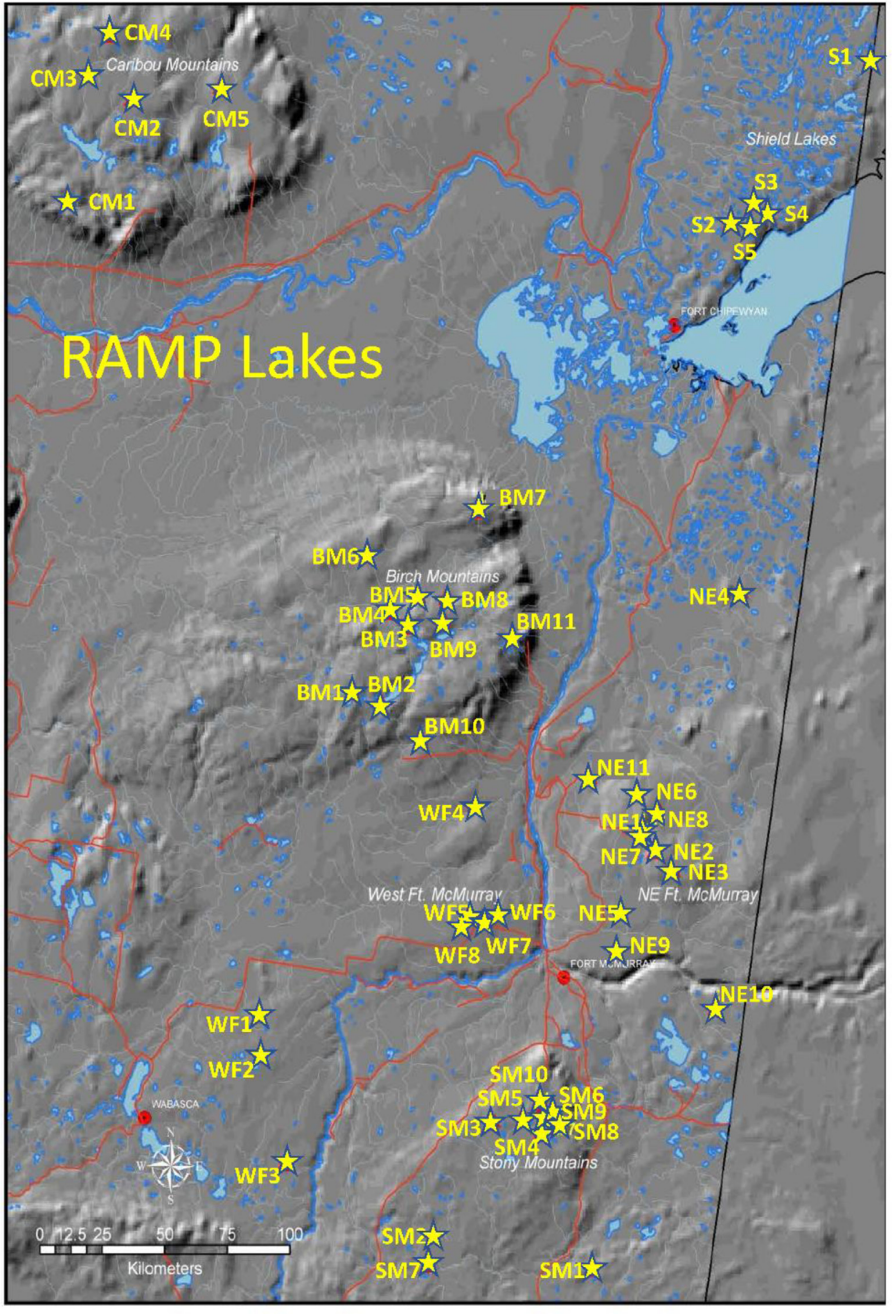
Map showing location of RAMP Lakes within the Alberta Oil Sands region. Note that topographic relief is exaggerated to highlight the position of plateaus, lowlands and incised river channels.

Lake, watershed, landcover, climate, stable isotope data (oxygen-18 and deuterium), water balance data, and Mann-Kendall statistics are provided from a program of hydrological and geochemical monitoring of 50 lakes in the oil sands region of northeastern Alberta over a 16-year period, during 2002–2017 (Fig. 1, Table 1, Table 2, Table 3, Table 4, Table 5, Table 6, Table 7, Table 8, Table 9, Table 10, Table 11, Table 12, Table 13, RAMPlakesWY.xlsx). Water sampling and analysis was supported by the University of Victoria, InnoTech Alberta, and Alberta Environment and Parks and its predecessors, and was designed to provide original data complimentary to geochemical characterization of lake-watershed systems for critical loads assessment.

**Table 1:** Lake, watershed and land cover data for RAMP sites, northeastern Alberta. Note that BFXC is bog forest permafrost collapse scar.

Lake No.	Lake ID	Lat	Long	Lake Area (m^2^)	Drainage Basin Area (m^2^)	Watershed Area (m^2^)	Volume (m^3^)	Max. Depth (m)	Mean Depth (m)	Drift thickness (m)	Distance to Buried Channel (m)	Elev. (masl)	Bog (%)	Fen (%)	Upland (%)	Open Water (%)	Permafrost (%)	BFXC (%)
1	NE1	57.15	−110.85	652,300	16,750,889	16,098,589	783,100	1.83	1.20	20.0	62,211	350	0.026	0.793	0.130	0.003	0.000	0.000
2	NE2	57.09	−110.75	336,700	15,130,803	14,794,103	427,900	1.83	1.27	26.1	19,955	483	0.012	0.504	0.469	0.000	0.000	0.000
3	NE3	57.05	−110.59	1,162,400	23,981,273	22,818,873	713,500	1.22	0.61	11.0	27,417	579	0.052	0.814	0.079	0.001	0.052	0.047
4	NE4	57.96	−110.40	581,800	3,173,982	2,592,182	842,500	2.13	1.45	0.0	7610	477	0.030	0.854	0.000	0.031	0.019	0.017
5	NE5	56.89	−110.90	1,894,900	7,320,388	5,425,488	1,731,200	1.83	0.91	29.9	90	719	0.180	0.687	0.074	0.018	0.253	0.116
6	NE6	57.27	−110.90	372,900	8,340,443	7,967,543	327,800	1.39	0.88	241.3	929	721	0.038	0.868	0.030	0.003	0.003	0.003
7	NE7	57.15	−110.86	111,900	5,910,314	5,798,414	112,300	2	1.00	92.1	11,872	720	0.004	0.695	0.244	0.000	0.000	0.000
8	NE8	57.23	−110.75	114,600	820,044	705,444	92,100	1.22	0.80	97.5	13,212	724	0.451	0.518	0.000	0.000	0.492	0.116
9	NE9	56.77	−110.91	3,154,800	11,210,595	8,055,795	3,517,800	1.83	1.12	0.1	2848	608	0.093	0.740	0.041	0.011	0.081	0.081
10	NE10	56.64	−110.20	4,188,000	17,090,907	12,902,907	3,227,700	1.5	0.77	9.5	25,253	787	0.035	0.539	0.320	0.003	0.007	0.007
11	NE11	57.29	−111.24	5,753,200	77,174,095	71,420,895	7,614,500	3.5	1.32	37.5	25,142	721	0.005	0.689	0.119	0.022	0.000	0.000
12	SM1	55.76	−110.76	2,369,500	9,610,510	7,241,010	1,594,200	1.83	0.67	3.4	27,803	685	0.001	0.501	0.212	0.045	0.000	0.000
13	SM2	55.79	−111.83	1,973,800	15,355,655	13,381,855	1,126,100	1.22	0.57	227.1	854	657	0.003	0.788	0.060	0.029	0.000	0.000
14	SM3	56.20	−111.37	1,861,300	7,391,411	5,530,111	2,691,700	3.05	1.45	156.8	9506	678	0.047	0.581	0.186	0.027	0.000	0.000
15	SM4	56.15	−111.23	525,600	11,740,623	11,215,023	371,100	1.22	0.71	190.7	3537	726	0.011	0.693	0.167	0.018	0.000	0.000
16	SM5	56.17	−111.55	1,061,000	3,670,195	2,609,195	1,219,500	1.83	1.15	153.2	12,954	757	0.012	0.635	0.195	0.071	0.000	0.000
17	SM6	56.22	−111.17	699,200	13,060,693	12,361,493	617,900	1.52	0.88	147.6	11,265	661	0.022	0.637	0.167	0.033	0.000	0.000
18	SM7	55.68	−111.83	1,476,100	6,940,368	5,464,268	1,885,700	3	1.28	158.5	8040	586	0.001	0.239	0.537	0.002	0.000	0.000
19	SM8	56.21	−111.20	1,912,500	9,630,511	7,718,011	1,694,600	2.5	0.89	147.9	6436	869	0.076	0.787	0.000	0.025	0.000	0.000
20	SM9	56.22	−111.25	1,071,400	8,280,439	7,209,039	608,000	1.2	0.57	125.0	22,255	903	0.128	0.722	0.000	0.050	0.000	0.000
21	SM10	56.26	−111.26	1,352,100	18,180,965	16,828,865	933,700	1.22	0.69	85.0	15,621	904	0.057	0.644	0.116	0.013	0.000	0.000
22	WF1	56.35	−113.18	3,203,400	10,430,554	7,227,154	1,874,800	1.22	0.59	0.0	20,741	885	0.044	0.647	0.200	0.010	0.000	0.000
23	WF2	56.24	−113.14	755,100	4,300,228	3,545,128	707,900	1.8	0.94	0.0	17,509	847	0.014	0.663	0.286	0.000	0.000	0.000
24	WF3	55.91	−112.86	2,163,500	51,552,736	49,389,236	2,090,700	2	0.97	45.6	25,379	590	0.016	0.902	0.011	0.000	0.000	0.000
25	WF4	57.15	−111.98	34,200	1,790,600	1,756,400	28,600	1.5	0.84	45.3	32,471	597	0.007	0.388	0.515	0.075	0.000	0.000
26	WF5	56.80	−111.92	234,500	5,040,267	4,805,767	176,700	1.22	0.75	19.3	34,853	652	0.035	0.485	0.451	0.000	0.000	0.000
27	WF6	56.81	−111.72	182,300	4,190,222	4,007,922	177,500	1.52	0.97	0.0	96,768	571	0.024	0.333	0.415	0.120	0.016	0.016
28	WF7	56.78	−111.79	85,000	1,590,084	1,505,084	67,500	1.22	0.79	11.6	27,937	590	0.036	0.314	0.606	0.000	0.000	0.000
29	WF8	56.77	−111.95	2,025,000	23,081,225	21,056,225	1,457,700	1.52	0.72	63.4	31,516	478	0.009	0.818	0.128	0.002	0.000	0.000
30	BM1	57.41	−112.93	17,029,700	58,723,117	41,693,417	98,076,200	9.14	5.76	98.3	19,562	334	0.460	0.133	0.134	0.004	0.470	0.378
31	BM2	57.42	−112.69	43,974,800	165,548,786	121,573,986	454,190,300	27.43	10.33	192.7	3732	568	0.429	0.149	0.130	0.009	0.429	0.255
32	BM3	57.65	−112.62	965,600	29,751,579	28,785,979	1,333,700	4.57	1.38	43.5	21,478	671	0.442	0.154	0.332	0.008	0.439	0.170
33	BM4	57.69	−112.74	4,264,100	37,331,982	33,067,882	1,828,200	1.22	0.43	19.4	29,666	722	0.616	0.060	0.184	0.012	0.615	0.251
34	BM5	57.76	−112.58	2,636,900	30,591,623	27,954,723	1,204,300	1.22	0.46	0.5	41,186	717	0.481	0.039	0.388	0.007	0.481	0.108
35	BM6	57.85	−112.97	1,290,200	13,670,726	12,380,526	639,900	0.91	0.50	2.5	50,456	721	0.802	0.097	0.005	0.000	0.854	0.802
36	BM7	58.06	−112.27	676,900	4,660,247	3,983,347	446,000	1.5	0.66	0.0	56,452	666	0.159	0.762	0.000	0.000	0.184	0.159
37	BM8	57.77	−112.40	1,215,100	32,491,725	31,276,625	1,358,900	1.83	1.12	1.7	49,261	625	0.161	0.618	0.179	0.006	0.167	0.026
38	BM9	57.70	−112.38	3,484,800	33,261,765	29,776,965	11,147,600	10.67	3.20	28.5	30,040	557	0.005	0.874	0.012	0.002	0.000	0.000
39	BM10	57.31	−112.40	393,700	5,150,273	4,756,573	145,600	1.5	0.37	63.7	3883	359	0.549	0.045	0.252	0.006	0.552	0.145
40	BM11	57.69	−111.91	55,000	570,030	515,030	13,100	5	0.24	130.8	31,924	510	0.032	0.569	0.369	0.000	0.000	0.000
41	CM1	58.77	−115.44	1,600,400	24,111,279	22,510,879	10,332,000	8.5	6.46	56.4	12,105	497	0.652	0.042	0.208	0.016	0.652	0.423
42	CM2	59.13	−115.13	9,550,300	46,772,483	37,222,183	27,318,000	6	2.86	70.5	27,362	517	0.657	0.128	0.012	0.018	0.750	0.574
43	CM3	59.19	−115.46	2,300,100	27,951,483	25,651,383	4,030,800	1.5	1.75	80.7	31,688	512	0.874	0.022	0.004	0.011	0.880	0.874
44	CM4	59.31	−115.35	2,627,800	38,052,019	35,424,219	21,733,200	16	8.27	0.0	21,927	745	0.788	0.084	0.001	0.014	0.854	0.762
45	CM5	59.24	−114.53	552,300	2,780,148	2,227,848	865,200	1.5	1.57	0.0	10,538	790	0.764	0.079	0.011	0.010	0.764	0.593
46	S1	59.72	−110.02	3,404,900	13,398,600	9,993,700	22,492,400	27.43	6.61	0.0	49,164	318	0.001	0.310	0.472	0.002	0.000	0.000
47	S2	59.12	−110.83	1,025,200	112,585,975	111,560,775	3,607,000	12.19	3.52	0.0	82,898	249	0.001	0.507	0.379	0.044	0.001	0.001
48	S3	59.19	−110.68	1,447,900	37,892,011	36,444,111	4,842,000	10.67	3.34	0.0	95,221	288	0.007	0.436	0.455	0.006	0.007	0.007
49	S4	59.17	−110.57	1,416,300	114,646,084	113,229,784	5,644,000	9.14	3.99	0.0	100,295	264	0.000	0.565	0.313	0.006	0.000	0.000
50	S5	59.13	−110.69	316,700	4,477,400	4,160,700	312,800	8.53	0.99	0.0	76,061	322	0.000	0.373	0.564	0.000	0.000	0.000

Lake and watershed parameters for the monitored lakes (Table 1), including lake area, drainage basin area, watershed area, and lake elevation were acquired using 1:50:000 raster-format digital elevation model (DEM) data from Canada National Topographic Series (NTS) map sheets according to pre-established protocols [2]. Vector-format hydrometric data were obtained from the Canadian National Hydro Network data obtained from the GeoBase portal http://www.geobase.com [3]. Watershed delineation utilized the ArcGIS program applying the ArcHYDRO tools, aided by preprocessing to fill small DEM sinks. Each individual watershed was delineated upstream of a lake outlet determined by hydrographic and elevation datasets. Lake and watershed areas were calculated based on equal area projections, and lake volumes, maximum depths, and mean depths were estimated based on bathymetric surveys conducted by Alberta Environment and Parks and its predecessors, mainly prior to 2005. Drift thickness and distance to buried channels for each lake were calculated in ArcGIS based on geological and hydrostratigraphic data layers obtained from the Alberta Geological Survey web portal (https://ags.aer.ca/data-maps-models/digital-data). Detailed land cover classification mapping and assessment of permafrost conditions from 1:20,000 air photos was carried out prior to 2005 by R. Bloise, Southern Illinois University (pers. Comm.) based on the Alberta Wetland Classification methodology [4] which was then used to estimate areal extent of these terrain types in each watershed, as summarized previously [5].

Climate data for the monitoring sites (Table 2, Table 3, Table 4, Table 5, Table 6, Table 7), including: (i) surface total precipitation (mm yr-1), (ii) 2-m relative humidity (%), (iii) surface evaporation (mm yr-1), and (iv) 2-m temperature (K), were.

**Table 2:** NARR climatology mean annual air temperature, interpolated for RAMP sites, northeastern Alberta.

Lake No.	Lake ID	T (deg C)															
		2002	2003	2004	2005	2006	2007	2008	2009	2010	2011	2012	2013	2014	2015	2016	2017
1	NE1	0.533	1.263	0.473	2.285	2.915	1.346	1.571	0.991	2.521	1.738	1.812	1.378	0.539	2.213	2.868	1.763
2	NE2	0.533	1.263	0.473	2.285	2.915	1.346	1.571	0.991	2.521	1.738	1.812	1.378	0.539	2.213	2.868	1.763
3	NE3	0.259	1.000	0.124	2.087	2.735	1.057	1.206	0.842	2.424	1.779	1.728	1.142	0.321	2.214	2.769	1.766
4	NE4	−0.040	0.941	−0.252	2.183	2.889	0.873	1.088	0.619	2.622	1.607	1.598	0.889	0.167	1.867	2.332	1.851
5	NE5	0.533	1.263	0.473	2.285	2.915	1.346	1.760	1.286	2.646	2.056	2.007	1.630	0.829	2.634	3.163	2.060
6	NE6	0.210	1.002	0.064	2.167	2.814	1.057	1.571	0.991	2.521	1.738	1.812	1.378	0.539	2.213	2.868	1.763
7	NE7	0.533	1.263	0.473	2.285	2.915	1.346	1.571	0.991	2.521	1.738	1.812	1.378	0.539	2.213	2.868	1.763
8	NE8	0.210	1.002	0.064	2.167	2.814	1.057	1.571	0.991	2.521	1.738	1.812	1.378	0.539	2.213	2.868	1.763
9	NE9	0.670	1.314	0.494	2.280	2.812	1.252	1.760	1.286	2.646	2.056	2.007	1.630	0.829	2.634	3.163	2.060
10	NE10	0.405	1.105	0.262	2.119	2.670	1.036	1.337	1.029	2.526	2.021	1.896	1.319	0.527	2.541	2.974	1.963
11	NE11	0.276	1.078	0.185	2.212	2.844	1.128	1.571	0.991	2.521	1.738	1.812	1.378	0.539	2.213	2.868	1.763
12	SM1	0.980	1.439	0.866	2.364	2.825	1.321	1.573	1.095	2.515	2.236	2.331	1.541	0.938	3.052	3.364	2.278
13	SM2	1.258	1.673	1.290	2.649	2.983	1.633	1.624	1.095	2.487	2.205	2.338	1.581	1.093	3.061	3.462	2.231
14	SM3	1.014	1.484	0.912	2.386	2.831	1.331	1.261	0.843	2.437	1.948	1.993	1.209	0.667	2.732	3.014	1.954
15	SM4	0.967	1.493	0.834	2.360	2.845	1.331	1.508	1.015	2.511	2.110	2.142	1.432	0.913	2.968	3.292	2.114
16	SM5	1.117	1.612	1.034	2.481	2.929	1.463	1.261	0.843	2.437	1.948	1.993	1.209	0.667	2.732	3.014	1.954
17	SM6	0.967	1.493	0.834	2.360	2.845	1.331	1.261	0.843	2.437	1.948	1.993	1.209	0.667	2.732	3.014	1.954
18	SM7	1.595	1.893	1.706	2.992	3.174	1.984	1.624	1.095	2.487	2.205	2.338	1.581	1.093	3.061	3.462	2.231
19	SM8	0.967	1.493	0.834	2.360	2.845	1.331	1.261	0.843	2.437	1.948	1.993	1.209	0.667	2.732	3.014	1.954
20	SM9	0.967	1.493	0.834	2.360	2.845	1.331	1.261	0.843	2.437	1.948	1.993	1.209	0.667	2.732	3.014	1.954
21	SM10	0.967	1.493	0.834	2.360	2.845	1.331	1.261	0.843	2.437	1.948	1.993	1.209	0.667	2.732	3.014	1.954
22	WF1	1.237	1.888	1.545	2.841	3.086	0.004	1.931	1.403	2.652	2.380	2.208	1.833	1.395	3.250	3.589	2.473
23	WF2	1.237	1.888	1.545	2.841	3.086	1.822	1.931	1.403	2.652	2.380	2.208	1.833	1.395	3.250	3.589	2.473
24	WF3	1.502	2.065	1.842	3.179	3.367	2.161	2.231	1.717	2.892	2.653	2.585	2.109	1.715	3.601	3.915	2.714
25	WF4	0.832	1.459	0.748	2.430	2.915	1.400	1.402	1.037	2.348	1.928	1.863	1.384	0.627	2.566	2.949	1.945
26	WF5	1.165	1.696	1.059	2.620	3.038	1.583	1.825	1.398	2.604	2.115	2.077	1.731	0.897	2.772	3.249	2.120
27	WF6	1.130	1.697	1.030	2.622	3.125	1.651	1.825	1.398	2.604	2.115	2.077	1.731	0.897	2.772	3.249	2.120
28	WF7	1.130	1.697	1.030	2.622	3.125	1.651	1.825	1.398	2.604	2.115	2.077	1.731	0.897	2.772	3.249	2.120
29	WF8	1.165	1.696	1.059	2.620	3.038	1.583	1.825	1.398	2.604	2.115	2.077	1.731	0.897	2.772	3.249	2.120
30	BM1	0.248	0.909	0.086	1.766	2.147	0.583	0.278	−0.006	1.614	0.925	0.875	0.205	−0.236	1.659	1.905	0.983
31	BM2	0.248	0.909	0.086	1.766	2.147	0.583	0.848	0.494	1.903	1.433	1.325	0.829	0.198	2.064	2.432	1.435
32	BM3	−0.352	0.404	−0.657	1.211	1.707	0.009	0.036	−0.280	1.421	0.694	0.715	−0.087	−0.540	1.332	1.630	0.727
33	BM4	−0.352	0.404	−0.657	1.211	1.707	0.009	0.036	−0.280	1.421	0.694	0.715	−0.087	−0.540	1.332	1.630	0.727
34	BM5	−0.352	0.404	−0.657	1.211	1.707	0.009	0.036	−0.280	1.421	0.694	0.715	−0.087	−0.540	1.332	1.630	0.727
35	BM6	−0.016	0.737	−0.270	1.497	1.983	0.320	1.326	1.188	2.695	1.961	1.970	1.454	1.016	2.827	2.833	2.626
36	BM7	−0.223	0.628	−0.560	1.524	2.206	0.312	−0.097	−0.402	1.424	0.632	0.655	−0.298	−0.755	1.150	1.439	0.678
37	BM8	−0.563	0.170	−0.929	1.057	1.587	−0.180	−0.097	−0.402	1.424	0.632	0.655	−0.298	−0.755	1.150	1.439	0.678
38	BM9	−0.563	0.170	−0.929	1.057	1.587	−0.180	−0.097	−0.402	1.424	0.632	0.655	−0.298	−0.755	1.150	1.439	0.678
39	BM10	0.128	0.785	−0.084	1.672	2.087	0.475	0.848	0.494	1.903	1.433	1.325	0.829	0.198	2.064	2.432	1.435
40	BM11	−0.479	0.224	−0.905	1.162	1.744	−0.089	−0.097	−0.402	1.424	0.632	0.655	−0.298	−0.755	1.150	1.439	0.678
41	CM1	−1.142	−0.283	−1.069	0.660	1.046	−0.671	0.988	0.963	1.978	1.344	1.302	1.473	1.136	2.668	2.724	2.221
42	CM2	−2.055	−1.395	−2.260	−0.356	0.045	−1.799	−1.729	−1.952	−0.010	−1.086	−0.889	−1.958	−1.933	−0.146	−0.021	−0.746
43	CM3	−1.840	−1.180	−1.971	−0.094	0.252	−1.546	−0.975	−1.138	0.659	−0.224	−0.268	−1.061	−1.138	0.608	0.666	0.180
44	CM4	−1.840	−1.180	−1.971	−0.094	0.252	−1.546	−1.830	−2.050	−0.120	−1.149	−1.124	−2.107	−2.078	−0.351	−0.197	−0.957
45	CM5	−1.949	−1.277	−2.231	−0.263	0.194	−1.733	−1.556	−1.653	0.216	−0.894	−0.692	−1.838	−1.819	−0.019	0.059	−0.444
46	S1	−1.816	−0.639	−2.008	0.551	1.708	−0.413	−0.743	−1.005	1.036	−0.370	0.173	−0.514	−1.359	−0.092	0.432	0.338
47	S2	−1.433	−0.124	−1.184	1.201	2.117	0.111	0.283	−0.231	1.591	0.463	1.001	0.567	−0.331	1.151	1.521	1.283
48	S3	−1.484	−0.240	−1.392	1.039	2.038	0.004	0.283	−0.231	1.591	0.463	1.001	0.567	−0.331	1.151	1.521	1.283
49	S4	−1.484	−0.240	−1.392	1.039	2.038	0.004	0.283	−0.231	1.591	0.463	1.001	0.567	−0.331	1.151	1.521	1.283
50	S5	−1.433	−0.124	−1.184	1.201	2.117	0.111	0.283	−0.231	1.591	0.463	1.001	0.567	−0.331	1.151	1.521	1.283

**Table 3:** NARR climatology flux-weighted air temperature, interpolated for RAMP sites, northeastern Alberta. This is our best estimate of average lake evaporation temperature.

Lake No.	Lake ID	T fw (deg C)															
		2002	2003	2004	2005	2006	2007	2008	2009	2010	2011	2012	2013	2014	2015	2016	2017
1	NE1	9.241	12.173	11.019	11.639	13.069	11.780	12.391	11.783	13.234	12.912	13.858	12.575	12.821	13.127	13.880	13.393
2	NE2	9.241	12.173	11.019	11.639	13.069	11.780	12.391	11.783	13.234	12.912	13.858	12.575	12.821	13.127	13.880	13.393
3	NE3	8.771	11.516	10.254	10.965	12.404	10.964	11.327	10.850	12.404	12.111	12.913	11.504	11.805	12.204	13.003	12.486
4	NE4	9.857	11.684	9.860	11.327	12.967	11.157	11.590	10.768	12.718	12.460	13.673	11.665	12.311	12.379	12.862	12.946
5	NE5	9.241	12.173	11.019	11.639	13.069	11.780	12.328	11.773	13.158	12.939	13.890	12.819	12.875	13.003	14.130	13.639
6	NE6	9.373	11.869	10.740	11.456	13.031	11.490	12.391	11.783	13.234	12.912	13.858	12.575	12.821	13.127	13.880	13.393
7	NE7	9.241	12.173	11.019	11.639	13.069	11.780	12.391	11.783	13.234	12.912	13.858	12.575	12.821	13.127	13.880	13.393
8	NE8	9.373	11.869	10.740	11.456	13.031	11.490	12.391	11.783	13.234	12.912	13.858	12.575	12.821	13.127	13.880	13.393
9	NE9	8.283	11.555	10.391	11.025	12.390	11.102	12.328	11.773	13.158	12.939	13.890	12.819	12.875	13.003	14.130	13.639
10	NE10	8.349	11.456	10.347	11.053	12.414	11.029	11.359	10.780	12.323	12.128	13.044	11.870	11.936	12.160	13.280	12.765
11	NE11	9.668	12.090	11.025	11.670	13.242	11.776	12.391	11.783	13.234	12.912	13.858	12.575	12.821	13.127	13.880	13.393
12	SM1	7.765	11.175	10.003	10.581	11.942	10.849	11.352	10.709	12.326	12.210	13.060	11.917	12.106	12.128	13.283	12.630
13	SM2	7.465	10.798	9.661	10.134	11.357	10.462	10.470	9.688	11.193	11.091	12.045	10.939	11.174	11.233	12.342	11.586
14	SM3	7.522	10.689	9.615	10.158	11.388	10.434	10.170	9.084	11.026	10.811	11.665	10.513	10.877	10.757	12.054	11.301
15	SM4	7.511	10.741	9.618	10.189	11.445	10.434	10.396	9.383	11.079	10.964	11.970	10.679	11.070	11.115	12.229	11.335
16	SM5	7.684	10.827	9.769	10.261	11.543	10.515	10.170	9.084	11.026	10.811	11.665	10.513	10.877	10.757	12.054	11.301
17	SM6	7.511	10.741	9.618	10.189	11.445	10.434	10.170	9.084	11.026	10.811	11.665	10.513	10.877	10.757	12.054	11.301
18	SM7	7.186	10.985	9.762	10.217	11.544	10.666	10.470	9.688	11.193	11.091	12.045	10.939	11.174	11.233	12.342	11.586
19	SM8	7.511	10.741	9.618	10.189	11.445	10.434	10.170	9.084	11.026	10.811	11.665	10.513	10.877	10.757	12.054	11.301
20	SM9	7.511	10.741	9.618	10.189	11.445	10.434	10.170	9.084	11.026	10.811	11.665	10.513	10.877	10.757	12.054	11.301
21	SM10	7.511	10.741	9.618	10.189	11.445	10.434	10.170	9.084	11.026	10.811	11.665	10.513	10.877	10.757	12.054	11.301
22	WF1	9.281	11.466	10.712	10.868	12.165	11.387	11.218	10.468	11.871	11.567	12.754	11.670	11.903	12.012	12.856	12.190
23	WF2	9.281	11.466	10.712	10.868	12.165	11.107	11.218	10.468	11.871	11.567	12.754	11.670	11.903	12.012	12.856	12.190
24	WF3	9.617	11.754	10.855	11.092	12.371	11.407	11.596	10.857	12.107	11.963	12.983	12.053	12.264	12.377	13.285	12.515
25	WF4	9.420	12.258	11.213	11.696	13.135	11.802	11.615	11.002	12.459	12.208	13.184	12.076	12.508	12.370	13.379	12.920
26	WF5	8.988	12.069	11.102	11.465	12.861	11.643	12.387	11.741	13.173	12.948	13.889	12.852	13.098	13.160	14.248	13.724
27	WF6	9.441	12.513	11.486	11.877	13.287	12.097	12.387	11.741	13.173	12.948	13.889	12.852	13.098	13.160	14.248	13.724
28	WF7	9.441	12.513	11.486	11.877	13.287	12.097	12.387	11.741	13.173	12.948	13.889	12.852	13.098	13.160	14.248	13.724
29	WF8	8.988	12.069	11.102	11.465	12.861	11.643	12.387	11.741	13.173	12.948	13.889	12.852	13.098	13.160	14.248	13.724
30	BM1	8.333	10.957	10.011	10.422	11.743	10.502	9.984	9.133	10.690	10.398	11.688	10.245	10.932	10.856	11.583	11.101
31	BM2	8.333	10.957	10.011	10.422	11.743	10.502	10.898	10.202	11.753	11.430	12.522	11.312	11.814	11.660	12.576	12.293
32	BM3	7.972	9.797	8.806	9.512	10.859	9.447	9.683	8.945	10.474	10.130	11.419	10.095	10.554	10.376	11.297	11.122
33	BM4	7.972	9.797	8.806	9.512	10.859	9.447	9.683	8.945	10.474	10.130	11.419	10.095	10.554	10.376	11.297	11.122
34	BM5	7.972	9.797	8.806	9.512	10.859	9.447	9.683	8.945	10.474	10.130	11.419	10.095	10.554	10.376	11.297	11.122
35	BM6	8.401	10.065	9.271	9.928	11.113	9.839	11.841	10.981	12.801	12.578	13.790	12.192	13.049	13.001	13.479	12.950
36	BM7	8.629	10.620	9.402	10.298	11.763	10.229	9.252	8.242	10.062	9.565	11.027	9.683	10.234	9.953	11.010	10.673
37	BM8	7.339	9.425	8.280	9.153	10.644	9.045	9.252	8.242	10.062	9.565	11.027	9.683	10.234	9.953	11.010	10.673
38	BM9	7.339	9.425	8.280	9.153	10.644	9.045	9.252	8.242	10.062	9.565	11.027	9.683	10.234	9.953	11.010	10.673
39	BM10	7.820	10.627	9.578	10.127	11.536	10.143	10.898	10.202	11.753	11.430	12.522	11.312	11.814	11.660	12.576	12.293
40	BM11	7.133	9.417	8.166	9.219	10.770	9.040	9.252	8.242	10.062	9.565	11.027	9.683	10.234	9.953	11.010	10.673
41	CM1	8.217	9.550	9.319	9.495	10.637	9.317	12.973	12.537	13.867	13.727	14.778	13.359	14.276	14.600	14.728	14.446
42	CM2	5.631	7.346	6.671	7.417	8.972	7.166	7.495	6.146	7.975	7.453	8.608	7.655	8.500	8.263	8.974	8.281
43	CM3	6.011	7.660	7.158	7.760	9.236	7.560	9.293	8.284	9.660	9.549	11.191	9.519	10.749	10.149	10.608	10.175
44	CM4	6.011	7.660	7.158	7.760	9.236	7.560	7.577	6.274	7.983	7.604	9.292	7.794	8.791	8.461	9.088	8.394
45	CM5	5.991	7.715	6.946	7.757	9.422	7.503	8.476	7.272	9.084	8.661	10.167	8.725	9.537	9.387	9.931	9.373
46	S1	9.227	11.354	9.487	10.792	12.703	10.733	11.302	10.531	12.766	11.727	13.230	11.572	11.981	12.015	12.279	12.073
47	S2	10.331	12.152	10.496	11.723	13.493	11.796	11.968	11.427	13.350	12.685	13.886	12.198	12.580	12.652	13.342	13.169
48	S3	9.879	11.833	10.157	11.376	13.121	11.387	11.968	11.427	13.350	12.685	13.886	12.198	12.580	12.652	13.342	13.169
49	S4	9.879	11.833	10.157	11.376	13.121	11.387	11.968	11.427	13.350	12.685	13.886	12.198	12.580	12.652	13.342	13.169
50	S5	10.331	12.152	10.496	11.723	13.493	11.796	11.968	11.427	13.350	12.685	13.886	12.198	12.580	12.652	13.342	13.169

**Table 4:** NARR climatology mean annual relative humidity, interpolated for RAMP sites, northeastern Alberta.

Lake No.	Lake ID	h															
		2002	2003	2004	2005	2006	2007	2008	2009	2010	2011	2012	2013	2014	2015	2016	2017
1	NE1	0.703	0.758	0.776	0.779	0.776	0.778	0.745	0.751	0.739	0.743	0.773	0.777	0.764	0.742	0.785	0.780
2	NE2	0.702	0.758	0.776	0.779	0.776	0.778	0.745	0.751	0.739	0.743	0.773	0.777	0.764	0.742	0.785	0.780
3	NE3	0.702	0.759	0.773	0.776	0.775	0.775	0.749	0.752	0.740	0.741	0.776	0.780	0.771	0.735	0.784	0.776
4	NE4	0.703	0.750	0.757	0.759	0.753	0.762	0.729	0.750	0.721	0.728	0.756	0.761	0.748	0.727	0.771	0.743
5	NE5	0.702	0.758	0.776	0.779	0.776	0.778	0.756	0.751	0.746	0.748	0.780	0.786	0.771	0.741	0.787	0.783
6	NE6	0.703	0.759	0.771	0.775	0.770	0.774	0.745	0.751	0.739	0.743	0.773	0.777	0.764	0.742	0.785	0.780
7	NE7	0.703	0.758	0.776	0.779	0.776	0.778	0.745	0.751	0.739	0.743	0.773	0.777	0.764	0.742	0.785	0.780
8	NE8	0.703	0.759	0.771	0.775	0.770	0.774	0.745	0.751	0.739	0.743	0.773	0.777	0.764	0.742	0.785	0.780
9	NE9	0.702	0.758	0.779	0.780	0.783	0.782	0.756	0.751	0.746	0.748	0.780	0.786	0.771	0.741	0.787	0.783
10	NE10	0.701	0.760	0.780	0.782	0.785	0.782	0.758	0.753	0.744	0.743	0.780	0.787	0.778	0.735	0.787	0.779
11	NE11	0.704	0.759	0.773	0.776	0.771	0.776	0.745	0.751	0.739	0.743	0.773	0.777	0.764	0.742	0.785	0.780
12	SM1	0.703	0.755	0.776	0.775	0.779	0.782	0.758	0.751	0.749	0.743	0.766	0.781	0.768	0.720	0.781	0.769
13	SM2	0.707	0.751	0.772	0.767	0.776	0.782	0.760	0.740	0.743	0.746	0.759	0.785	0.773	0.721	0.783	0.774
14	SM3	0.704	0.752	0.774	0.772	0.777	0.782	0.755	0.743	0.739	0.738	0.762	0.781	0.771	0.715	0.779	0.767
15	SM4	0.704	0.752	0.774	0.772	0.777	0.782	0.754	0.738	0.740	0.740	0.761	0.781	0.771	0.713	0.778	0.767
16	SM5	0.705	0.751	0.775	0.771	0.776	0.784	0.755	0.743	0.739	0.738	0.762	0.781	0.771	0.715	0.779	0.767
17	SM6	0.704	0.752	0.774	0.772	0.777	0.782	0.755	0.743	0.739	0.738	0.762	0.781	0.771	0.715	0.779	0.767
18	SM7	0.708	0.749	0.767	0.760	0.773	0.777	0.760	0.740	0.743	0.746	0.759	0.785	0.773	0.721	0.783	0.774
19	SM8	0.704	0.752	0.774	0.772	0.777	0.782	0.755	0.743	0.739	0.738	0.762	0.781	0.771	0.715	0.779	0.767
20	SM9	0.704	0.752	0.774	0.772	0.777	0.782	0.755	0.743	0.739	0.738	0.762	0.781	0.771	0.715	0.779	0.767
21	SM10	0.704	0.752	0.774	0.772	0.777	0.782	0.755	0.743	0.739	0.738	0.762	0.781	0.771	0.715	0.779	0.767
22	WF1	0.714	0.752	0.776	0.767	0.776	0.766	0.759	0.739	0.737	0.749	0.766	0.785	0.767	0.718	0.780	0.768
23	WF2	0.714	0.752	0.776	0.767	0.776	0.787	0.759	0.739	0.737	0.749	0.766	0.785	0.767	0.718	0.780	0.768
24	WF3	0.712	0.753	0.777	0.765	0.777	0.786	0.759	0.736	0.737	0.750	0.763	0.786	0.770	0.716	0.780	0.771
25	WF4	0.707	0.758	0.778	0.779	0.779	0.785	0.756	0.745	0.738	0.744	0.771	0.779	0.768	0.730	0.783	0.778
26	WF5	0.706	0.756	0.778	0.778	0.780	0.786	0.761	0.752	0.746	0.751	0.779	0.786	0.776	0.740	0.789	0.788
27	WF6	0.705	0.761	0.783	0.782	0.784	0.789	0.761	0.752	0.746	0.751	0.779	0.786	0.776	0.740	0.789	0.788
28	WF7	0.706	0.761	0.783	0.782	0.784	0.789	0.761	0.752	0.746	0.751	0.779	0.786	0.776	0.740	0.789	0.788
29	WF8	0.707	0.756	0.778	0.778	0.780	0.786	0.761	0.752	0.746	0.751	0.779	0.786	0.776	0.740	0.789	0.788
30	BM1	0.711	0.752	0.771	0.773	0.775	0.784	0.763	0.748	0.742	0.748	0.766	0.785	0.768	0.727	0.790	0.768
31	BM2	0.710	0.752	0.771	0.773	0.775	0.784	0.758	0.745	0.739	0.747	0.769	0.780	0.766	0.731	0.784	0.775
32	BM3	0.710	0.752	0.768	0.775	0.770	0.782	0.759	0.751	0.740	0.745	0.762	0.780	0.767	0.730	0.789	0.770
33	BM4	0.710	0.752	0.768	0.775	0.770	0.782	0.759	0.751	0.740	0.745	0.762	0.780	0.767	0.730	0.789	0.770
34	BM5	0.709	0.752	0.768	0.775	0.770	0.782	0.759	0.751	0.740	0.745	0.762	0.780	0.767	0.730	0.789	0.770
35	BM6	0.710	0.750	0.768	0.776	0.771	0.785	0.753	0.747	0.730	0.738	0.750	0.770	0.747	0.716	0.780	0.743
36	BM7	0.709	0.750	0.766	0.774	0.764	0.778	0.749	0.746	0.732	0.735	0.755	0.776	0.762	0.723	0.786	0.764
37	BM8	0.709	0.753	0.769	0.773	0.768	0.780	0.749	0.746	0.732	0.735	0.755	0.776	0.762	0.723	0.786	0.764
38	BM9	0.709	0.753	0.769	0.773	0.768	0.780	0.749	0.746	0.732	0.735	0.755	0.776	0.762	0.723	0.786	0.764
39	BM10	0.709	0.751	0.769	0.771	0.772	0.781	0.758	0.745	0.739	0.747	0.769	0.780	0.766	0.731	0.784	0.775
40	BM11	0.707	0.753	0.768	0.772	0.765	0.777	0.749	0.746	0.732	0.735	0.755	0.776	0.762	0.723	0.786	0.764
41	CM1	0.703	0.748	0.742	0.767	0.769	0.780	0.774	0.756	0.765	0.763	0.761	0.781	0.748	0.716	0.781	0.749
42	CM2	0.706	0.748	0.739	0.763	0.765	0.774	0.752	0.748	0.738	0.743	0.731	0.775	0.742	0.720	0.778	0.752
43	CM3	0.704	0.750	0.736	0.762	0.766	0.774	0.755	0.750	0.741	0.745	0.736	0.767	0.740	0.726	0.775	0.752
44	CM4	0.705	0.750	0.736	0.762	0.766	0.774	0.755	0.750	0.741	0.744	0.739	0.776	0.745	0.728	0.780	0.757
45	CM5	0.709	0.743	0.737	0.761	0.762	0.772	0.744	0.741	0.732	0.736	0.726	0.769	0.736	0.716	0.776	0.746
46	S1	0.706	0.756	0.759	0.767	0.756	0.762	0.733	0.755	0.741	0.748	0.752	0.758	0.739	0.741	0.777	0.752
47	S2	0.706	0.757	0.753	0.766	0.759	0.769	0.734	0.755	0.740	0.743	0.747	0.757	0.734	0.724	0.773	0.737
48	S3	0.706	0.756	0.756	0.766	0.757	0.766	0.734	0.755	0.740	0.743	0.747	0.757	0.734	0.724	0.773	0.737
49	S4	0.706	0.756	0.756	0.766	0.757	0.766	0.734	0.755	0.740	0.743	0.747	0.757	0.734	0.724	0.773	0.737
50	S5	0.706	0.757	0.753	0.766	0.759	0.769	0.734	0.755	0.740	0.743	0.747	0.757	0.734	0.724	0.773	0.737

**Table 5:** NARR climatology flux-weighted relative humidity, interpolated for RAMP sites, northeastern Alberta. This is our best estimate of average relative humidity of the atmosphere during the lake evaporation season.

Lake No.	Lake ID	h fw															
		2002	2003	2004	2005	2006	2007	2008	2009	2010	2011	2012	2013	2014	2015	2016	2017
1	NE1	0.660	0.685	0.691	0.703	0.706	0.709	0.665	0.672	0.657	0.665	0.689	0.714	0.689	0.643	0.703	0.692
2	NE2	0.659	0.685	0.691	0.703	0.706	0.709	0.665	0.672	0.657	0.665	0.689	0.714	0.689	0.643	0.703	0.692
3	NE3	0.663	0.685	0.690	0.702	0.704	0.705	0.676	0.683	0.663	0.666	0.692	0.720	0.697	0.641	0.703	0.694
4	NE4	0.672	0.667	0.659	0.667	0.661	0.669	0.632	0.667	0.628	0.635	0.651	0.677	0.647	0.622	0.679	0.639
5	NE5	0.658	0.685	0.691	0.703	0.706	0.709	0.682	0.675	0.669	0.671	0.696	0.727	0.702	0.643	0.705	0.699
6	NE6	0.662	0.684	0.682	0.695	0.693	0.696	0.665	0.672	0.657	0.665	0.689	0.714	0.689	0.643	0.703	0.692
7	NE7	0.659	0.685	0.691	0.703	0.706	0.709	0.665	0.672	0.657	0.665	0.689	0.714	0.689	0.643	0.703	0.692
8	NE8	0.662	0.684	0.682	0.695	0.693	0.696	0.665	0.672	0.657	0.665	0.689	0.714	0.689	0.643	0.703	0.692
9	NE9	0.658	0.690	0.704	0.714	0.719	0.723	0.682	0.675	0.669	0.671	0.696	0.727	0.702	0.643	0.705	0.699
10	NE10	0.658	0.688	0.703	0.713	0.718	0.719	0.689	0.684	0.670	0.671	0.699	0.731	0.711	0.644	0.709	0.701
11	NE11	0.661	0.683	0.684	0.696	0.695	0.698	0.665	0.672	0.657	0.665	0.689	0.714	0.689	0.643	0.703	0.692
12	SM1	0.659	0.690	0.706	0.719	0.716	0.725	0.691	0.680	0.672	0.670	0.681	0.723	0.707	0.628	0.698	0.699
13	SM2	0.667	0.687	0.702	0.713	0.714	0.727	0.698	0.670	0.670	0.675	0.674	0.726	0.711	0.635	0.708	0.711
14	SM3	0.663	0.690	0.706	0.716	0.717	0.727	0.694	0.678	0.670	0.671	0.682	0.724	0.708	0.629	0.704	0.702
15	SM4	0.662	0.690	0.706	0.716	0.717	0.727	0.694	0.672	0.670	0.672	0.679	0.726	0.709	0.627	0.702	0.703
16	SM5	0.664	0.689	0.706	0.716	0.716	0.729	0.694	0.678	0.670	0.671	0.682	0.724	0.708	0.629	0.704	0.702
17	SM6	0.662	0.690	0.706	0.716	0.717	0.727	0.694	0.678	0.670	0.671	0.682	0.724	0.708	0.629	0.704	0.702
18	SM7	0.668	0.681	0.699	0.708	0.708	0.724	0.698	0.670	0.670	0.675	0.674	0.726	0.711	0.635	0.708	0.711
19	SM8	0.662	0.690	0.706	0.716	0.717	0.727	0.694	0.678	0.670	0.671	0.682	0.724	0.708	0.629	0.704	0.702
20	SM9	0.662	0.690	0.706	0.716	0.717	0.727	0.694	0.678	0.670	0.671	0.682	0.724	0.708	0.629	0.704	0.702
21	SM10	0.662	0.690	0.706	0.716	0.717	0.727	0.694	0.678	0.670	0.671	0.682	0.724	0.708	0.629	0.704	0.702
22	WF1	0.676	0.685	0.701	0.710	0.715	0.661	0.699	0.659	0.659	0.681	0.680	0.730	0.700	0.625	0.703	0.700
23	WF2	0.676	0.685	0.701	0.710	0.715	0.731	0.699	0.659	0.659	0.681	0.680	0.730	0.700	0.625	0.703	0.700
24	WF3	0.672	0.684	0.704	0.710	0.714	0.729	0.699	0.657	0.661	0.680	0.677	0.728	0.705	0.623	0.704	0.703
25	WF4	0.663	0.685	0.694	0.705	0.709	0.719	0.687	0.661	0.658	0.672	0.686	0.721	0.696	0.633	0.702	0.700
26	WF5	0.665	0.686	0.698	0.710	0.715	0.727	0.689	0.670	0.668	0.676	0.694	0.727	0.706	0.640	0.706	0.704
27	WF6	0.660	0.689	0.702	0.712	0.718	0.728	0.689	0.670	0.668	0.676	0.694	0.727	0.706	0.640	0.706	0.704
28	WF7	0.661	0.689	0.702	0.712	0.718	0.728	0.689	0.670	0.668	0.676	0.694	0.727	0.706	0.640	0.706	0.704
29	WF8	0.665	0.686	0.698	0.710	0.715	0.727	0.689	0.670	0.668	0.676	0.694	0.727	0.706	0.640	0.706	0.704
30	BM1	0.675	0.683	0.688	0.705	0.710	0.721	0.703	0.679	0.671	0.682	0.688	0.729	0.696	0.640	0.719	0.696
31	BM2	0.674	0.683	0.688	0.705	0.710	0.721	0.695	0.666	0.660	0.676	0.687	0.724	0.695	0.638	0.705	0.701
32	BM3	0.679	0.688	0.689	0.711	0.706	0.719	0.694	0.681	0.666	0.674	0.679	0.717	0.691	0.640	0.717	0.696
33	BM4	0.679	0.688	0.689	0.711	0.706	0.719	0.694	0.681	0.666	0.674	0.679	0.717	0.691	0.640	0.717	0.696
34	BM5	0.679	0.688	0.689	0.711	0.706	0.719	0.694	0.681	0.666	0.674	0.679	0.717	0.691	0.640	0.717	0.696
35	BM6	0.682	0.686	0.688	0.716	0.712	0.724	0.687	0.677	0.656	0.661	0.656	0.702	0.658	0.617	0.702	0.664
36	BM7	0.678	0.681	0.682	0.703	0.695	0.707	0.680	0.679	0.659	0.665	0.670	0.710	0.684	0.632	0.716	0.692
37	BM8	0.677	0.691	0.692	0.710	0.702	0.715	0.680	0.679	0.659	0.665	0.670	0.710	0.684	0.632	0.716	0.692
38	BM9	0.677	0.691	0.692	0.710	0.702	0.715	0.680	0.679	0.659	0.665	0.670	0.710	0.684	0.632	0.716	0.692
39	BM10	0.671	0.685	0.689	0.704	0.706	0.718	0.695	0.666	0.660	0.676	0.687	0.724	0.695	0.638	0.705	0.701
40	BM11	0.673	0.691	0.694	0.708	0.698	0.711	0.680	0.679	0.659	0.665	0.670	0.710	0.684	0.632	0.716	0.692
41	CM1	0.681	0.682	0.647	0.699	0.715	0.718	0.712	0.678	0.683	0.682	0.662	0.717	0.656	0.594	0.691	0.661
42	CM2	0.689	0.694	0.658	0.702	0.710	0.717	0.697	0.698	0.683	0.679	0.654	0.721	0.667	0.639	0.716	0.687
43	CM3	0.686	0.693	0.652	0.700	0.712	0.715	0.691	0.691	0.681	0.671	0.642	0.704	0.646	0.640	0.703	0.679
44	CM4	0.687	0.693	0.652	0.700	0.712	0.715	0.700	0.699	0.687	0.680	0.657	0.725	0.667	0.650	0.718	0.694
45	CM5	0.691	0.688	0.653	0.698	0.702	0.711	0.679	0.688	0.667	0.666	0.638	0.706	0.649	0.626	0.710	0.670
46	S1	0.681	0.669	0.653	0.663	0.653	0.653	0.624	0.661	0.626	0.647	0.642	0.666	0.626	0.621	0.682	0.648
47	S2	0.680	0.676	0.646	0.663	0.659	0.666	0.631	0.662	0.630	0.645	0.643	0.672	0.627	0.608	0.680	0.633
48	S3	0.681	0.674	0.651	0.664	0.657	0.661	0.631	0.662	0.630	0.645	0.643	0.672	0.627	0.608	0.680	0.633
49	S4	0.681	0.674	0.651	0.664	0.657	0.661	0.631	0.662	0.630	0.645	0.643	0.672	0.627	0.608	0.680	0.633
50	S5	0.680	0.676	0.646	0.663	0.659	0.666	0.631	0.662	0.630	0.645	0.643	0.672	0.627	0.608	0.680	0.633

**Table 6:** NARR climatology mean annual open-water evaporation, interpolated for RAMP sites, northeastern Alberta.

Lake No.	Lake ID	E(mm)															
		2002	2003	2004	2005	2006	2007	2008	2009	2010	2011	2012	2013	2014	2015	2016	2017
1	NE1	363	544	496	535	573	547	555	538	550	553	604	580	569	561	583	563
2	NE2	363	544	496	535	573	547	555	538	550	553	604	580	569	561	583	563
3	NE3	372	532	477	518	559	525	563	544	556	552	612	593	571	555	578	576
4	NE4	395	474	407	453	481	442	496	468	469	473	508	497	475	489	474	465
5	NE5	363	544	496	535	573	547	599	565	585	570	636	629	611	578	603	591
6	NE6	382	538	478	527	556	517	555	538	550	553	604	580	569	561	583	563
7	NE7	363	544	496	535	573	547	555	538	550	553	604	580	569	561	583	563
8	NE8	382	538	478	527	556	517	555	538	550	553	604	580	569	561	583	563
9	NE9	361	550	521	559	609	578	599	565	585	570	636	629	611	578	603	591
10	NE10	366	538	516	566	607	572	591	557	569	562	638	629	608	584	601	602
11	NE11	386	534	484	532	556	521	555	538	550	553	604	580	569	561	583	563
12	SM1	376	576	550	595	642	619	651	596	602	609	668	642	625	570	612	639
13	SM2	377	550	518	568	614	586	610	569	563	586	623	613	594	580	597	619
14	SM3	377	563	529	572	624	597	606	567	576	573	631	605	588	548	577	608
15	SM4	377	563	529	572	624	597	600	569	579	580	636	612	596	553	580	603
16	SM5	378	557	525	569	616	594	606	567	576	573	631	605	588	548	577	608
17	SM6	377	563	529	572	624	597	606	567	576	573	631	605	588	548	577	608
18	SM7	372	550	522	577	624	592	610	569	563	586	623	613	594	580	597	619
19	SM8	377	563	529	572	624	597	606	567	576	573	631	605	588	548	577	608
20	SM9	377	563	529	572	624	597	606	567	576	573	631	605	588	548	577	608
21	SM10	377	563	529	572	624	597	606	567	576	573	631	605	588	548	577	608
22	WF1	403	545	533	569	609	457	613	564	551	590	626	617	602	556	584	598
23	WF2	403	545	533	569	609	588	613	564	551	590	626	617	602	556	584	598
24	WF3	410	563	545	580	623	595	621	569	564	612	633	628	624	557	607	621
25	WF4	376	547	514	555	583	565	576	532	541	569	620	601	587	569	589	592
26	WF5	360	551	519	564	600	582	600	555	581	594	639	628	619	587	604	603
27	WF6	365	573	537	567	622	601	600	555	581	594	639	628	619	587	604	603
28	WF7	365	573	537	567	622	601	600	555	581	594	639	628	619	587	604	603
29	WF8	360	551	519	564	600	582	600	555	581	594	639	628	619	587	604	603
30	BM1	363	499	480	515	545	533	551	509	517	526	575	561	543	533	549	531
31	BM2	363	499	480	515	545	533	561	524	522	541	597	578	563	546	561	573
32	BM3	407	483	448	495	510	499	519	493	485	490	531	521	517	510	529	535
33	BM4	407	483	448	495	510	499	519	493	485	490	531	521	517	510	529	535
34	BM5	407	483	448	495	510	499	519	493	485	490	531	521	517	510	529	535
35	BM6	425	499	477	521	536	524	588	558	564	561	601	577	571	590	577	596
36	BM7	436	504	466	507	518	502	499	469	453	463	529	521	505	493	534	550
37	BM8	404	481	443	491	498	484	499	469	453	463	529	521	505	493	534	550
38	BM9	404	481	443	491	498	484	499	469	453	463	529	521	505	493	534	550
39	BM10	371	503	476	512	534	519	561	524	522	541	597	578	563	546	561	573
40	BM11	411	497	460	509	510	492	499	469	453	463	529	521	505	493	534	550
41	CM1	438	482	426	486	498	469	619	580	587	599	590	599	603	559	601	596
42	CM2	390	432	356	429	440	415	445	420	451	418	407	461	405	395	456	462
43	CM3	399	450	377	438	456	426	517	490	532	507	516	506	494	524	501	540
44	CM4	399	450	377	438	456	426	456	429	464	439	471	466	438	460	472	486
45	CM5	389	428	349	431	443	418	467	440	464	436	435	480	418	411	482	461
46	S1	425	474	417	452	481	456	475	442	456	484	515	511	475	470	481	509
47	S2	453	494	413	460	483	469	485	454	455	496	520	513	446	424	489	475
48	S3	436	481	416	455	477	457	485	454	455	496	520	513	446	424	489	475
49	S4	436	481	416	455	477	457	485	454	455	496	520	513	446	424	489	475
50	S5	453	494	413	460	483	469	485	454	455	496	520	513	446	424	489	475

**Table 7:** NARR climatology mean annual precipitation, interpolated for RAMP sites, northeastern Alberta.

Lake No.	Lake ID	P(mm)															
		2002	2003	2004	2005	2006	2007	2008	2009	2010	2011	2012	2013	2014	2015	2016	2017
1	NE1	398	605	580	530	604	576	641	567	575	493	805	567	481	429	642	526
2	NE2	398	605	580	530	604	578	641	567	575	493	805	567	481	429	642	526
3	NE3	401	596	561	539	579	571	642	569	568	496	789	568	488	440	624	542
4	NE4	417	627	476	572	545	523	537	582	506	463	667	529	428	464	668	462
5	NE5	398	605	580	530	604	576	680	558	606	500	803	611	526	437	656	557
6	NE6	413	613	546	541	556	538	641	567	575	493	805	567	481	429	642	526
7	NE7	398	605	580	530	604	578	641	567	575	493	805	567	481	429	642	526
8	NE8	413	613	546	541	556	538	641	567	575	493	805	567	481	429	642	526
9	NE9	386	567	602	544	638	605	680	558	606	500	803	611	526	437	656	557
10	NE10	384	561	608	560	632	599	640	545	575	499	750	608	520	452	626	554
11	NE11	405	597	556	526	555	537	641	567	575	493	805	567	481	429	642	526
12	SM1	388	553	604	567	709	682	628	519	578	570	682	658	581	480	675	642
13	SM2	377	533	589	548	683	673	612	468	570	558	627	641	561	458	720	628
14	SM3	386	564	598	536	678	664	638	511	594	524	715	626	566	430	654	586
15	SM4	386	564	598	536	678	655	604	493	563	520	662	615	547	429	645	591
16	SM5	389	574	604	551	683	671	638	511	594	524	715	626	566	430	654	586
17	SM6	386	564	598	536	678	655	638	511	594	524	715	626	566	430	654	586
18	SM7	389	504	612	559	693	673	612	468	570	558	627	641	561	458	720	628
19	SM8	386	564	598	536	678	664	638	511	594	524	715	626	566	430	654	586
20	SM9	386	564	598	536	678	655	638	511	594	524	715	626	566	430	654	586
21	SM10	386	564	598	536	678	655	638	511	594	524	715	626	566	430	654	586
22	WF1	400	557	610	561	649	434	635	443	536	599	633	643	520	440	726	556
23	WF2	400	557	610	561	649	679	635	443	536	599	633	643	520	440	726	556
24	WF3	396	549	662	603	685	711	626	450	562	626	642	682	540	441	776	603
25	WF4	412	562	595	504	599	591	641	490	566	540	743	592	526	400	637	523
26	WF5	419	576	604	532	635	650	659	493	600	548	762	615	555	404	662	548
27	WF6	417	630	652	567	698	669	686	531	616	529	793	622	557	417	681	559
28	WF7	417	630	652	567	698	669	659	493	600	548	762	615	555	404	662	548
29	WF8	419	576	604	532	635	644	659	493	600	548	762	615	555	404	662	548
30	BM1	407	520	551	456	573	551	590	476	521	506	634	569	485	407	641	447
31	BM2	407	520	551	456	573	574	586	489	521	502	662	561	487	401	632	480
32	BM3	394	487	475	429	495	509	540	490	505	439	570	497	450	393	611	453
33	BM4	394	487	475	429	495	509	540	490	505	439	570	497	450	393	611	453
34	BM5	394	487	475	429	495	509	540	490	505	439	570	497	450	393	611	453
35	BM6	398	501	493	443	527	520	568	512	546	433	538	512	458	429	666	474
36	BM7	417	507	486	446	490	511	527	521	520	424	581	506	420	399	651	460
37	BM8	407	485	467	425	479	502	530	500	498	436	593	497	436	385	615	467
38	BM9	407	485	467	425	479	502	530	500	498	436	593	497	436	385	615	467
39	BM10	409	510	534	452	541	536	586	489	521	502	662	561	487	401	632	480
40	BM11	418	498	483	438	482	493	530	500	498	436	593	497	436	385	615	467
41	CM1	416	459	409	467	533	515	619	561	511	457	489	559	457	447	575	487
42	CM2	433	413	354	430	453	478	473	473	430	348	404	452	354	429	484	432
43	CM3	433	442	359	460	471	488	537	530	504	408	453	469	402	500	521	492
44	CM4	433	442	359	460	471	488	482	486	440	357	419	441	353	457	489	442
45	CM5	446	408	361	428	457	486	501	519	451	362	450	460	355	456	531	435
46	S1	374	424	361	455	392	434	453	390	393	419	447	432	402	418	541	388
47	S2	386	409	371	456	383	445	431	414	370	361	464	444	339	416	527	374
48	S3	383	413	369	457	379	434	431	414	370	361	464	444	339	416	527	374
49	S4	383	413	369	457	379	439	431	414	370	361	464	444	339	416	527	374
50	S5	386	409	371	456	383	445	431	414	370	361	464	444	339	416	527	374

Interpolated from the 32-km resolution North American Regional Reanalsis (NARR) monthly climatology [6] using the Grid Analysis Display System (GrADS) [7]. An evaporation flux-weighting protocol [8] was also applied to condition climate data to improve representativeness for assessment of isotope-based water balance, as used in numerous Canadian and international assessments [[9], [10], [11], [12], [13], [14], [15], [16], [17], [18], [19], [20]].

From 2008 to 2017, a dual-inlet Thermofisher Scientific Isotope Ratio Mass Spectrometer, Delta V interfaced with a Gasbench peripheral (for oxygen-18) and H-Device peripheral (for deuterium) was used for isotopic analysis [21,22]. Comparable protocols were employed to measure isotopic content during 2002–2007 [5]. Results are reported in “δ” notation in per mil (‰) relative to Vienna Standard Mean Ocean Water (V-SMOW), normalized on the SMOW/SLAP scale [23]. Analytical uncertainty, as estimated from standard deviation of repeats, is better than ±0.1‰ for δ^18^O and ±1‰ for δ^2^H. Raw isotopic data for lake water samples are provided in Table 8, Table 9 Mean values for each lake are summarized in Table 10 in comparison with interpolated estimates of isotope composition of precipitation and atmospheric moisture for each of the 50 sites. Monthly precipitation δ^18^O estimates were obtained for each lake/watershed location based on a protocol developed using empirically derived global relationships between latitude and elevation [24] fitted to regional precipitation data from the Canadian Network for Isotopes in Precipitation [25]. The δ^2^H composition of monthly precipitation was calculated assuming that precipitation would follow the relationship defined by the Global Meteoric Water Line (GMWL; [26]). Annual δ^18^O and δ^2^H in precipitation were then amount-weighted using monthly isotope data and NARR precipitation amounts. Isotope balance estimates of evaporation/inflow (Table 11) and water yield to lakes (Table 12) were based on a previously demonstrated model and protocols [12].

**Table 8:** Annual δ^18^O measurements as measured in late summer/early fall for RAMP lake water, northeastern Alberta.

Lake No.	Lake ID	δ^18^O_L_ (per mil)															
		2002	2003	2004	2005	2006	2007	2008	2009	2010	2011	2012	2013	2014	2015	2016	2017
1	NE1	−16.09	−15.19	−14.47	−15.82	−14.83	−13.30	−16.46	−15.39	−13.20	−11.78	−14.34	−16.08	−15.12	−13.44	−14.94	−14.03
2	NE2	−16.62	−15.23	−14.60	−15.85	−15.85	−13.79	−15.75	−15.62	−14.88	−12.63	−14.76	−15.45	−15.64	−12.43	−15.12	−13.69
3	NE3	−12.92	−13.12	−12.95	−14.58	−14.58	−10.73	−13.26	−13.24	−12.43	−11.34	−12.04	−14.37	−13.89	−11.46	−12.25	−12.33
4	NE4	−14.99	−14.18	−14.16	−15.64	−13.46	−12.35	−14.50	−15.08	−13.20	−13.60	−13.08	−14.68	−14.36	−13.05	−13.87	−13.07
5	NE5	−10.75	−11.48	−11.05	−11.33	−10.03	−7.97	−10.89	−11.72	−10.91	−9.85	−10.43	−12.84	−12.12	−10.25	−10.77	−10.71
6	NE6	−15.19	−14.19	−13.15	−15.54	−12.94	−14.89	−10.52	−14.42	−15.81	−13.72	−14.45	−14.12	−14.31	−13.16	−14.14	−14.18
7	NE7	−16.96	−15.84	−15.57	−16.28	−15.70	−15.85	−16.36	−15.88	−16.15	−13.79	−16.42	−16.58	−15.13	−13.53	−15.91	−15.03
8	NE8	−15.90	−14.55	−13.62	−15.44	−13.75	−13.12	−15.79	−15.01	−15.50	−13.04	−14.71	−15.45	−14.36	−11.68	−14.30	−13.06
9	NE9	−9.07	−9.09	−9.55	−9.82	−9.68	−7.83	−9.36	−10.91	−9.97	−9.13	−9.23	−11.16	−10.81	−8.93	−9.20	−9.16
10	NE10	−8.70	−8.13	−9.76	−10.52	−9.46	−8.97	−9.43	−10.94	−9.31	−8.66	−8.19	−10.47	−10.39	−8.11	−8.27	−9.01
11	NE11		−12.56	−12.31	−13.48	−11.16	−9.04	−11.74	−12.18	−10.91	−9.93	−9.42	−13.50	−13.64	−10.16	−10.46	−10.41
12	SM1	−8.37	−8.33	−9.28	−9.36	−8.26	−7.20	−10.08	−10.06	−9.61	−9.26	−7.86	−10.35	−10.00	−8.44	−8.07	−10.17
13	SM2	−7.15	−7.01	−8.88	−9.71	−8.53	−7.17	−9.64	−9.63	−9.40	−9.28	−8.71	−10.69	−9.82	−7.62	−8.29	−9.35
14	SM3	−9.62	−9.72	−9.88	−11.02	−10.08	−9.49	−10.46	−10.87	−10.64	−10.10	−9.43	−11.66	−11.60	−9.26	−8.92	−9.61
15	SM4	−10.00	−11.73	−11.26	−11.55	−11.36	−11.03	−12.07	−12.43	−12.15	−12.22	−11.41	−14.43	−13.61	−9.39	−11.57	−13.27
16	SM5	−9.88	−9.30	−9.71	−9.94	−9.55	−9.24	−11.33	−10.91	−10.97	−9.96	−8.71	−11.79	−11.63	−9.03	−8.60	−9.46
17	SM6	−10.32	−10.17	−10.91	−11.44	−10.91	−10.35	−11.46	−11.38	−11.09	−11.26	−10.84	−13.54	−12.71	−10.10	−9.46	−11.47
18	SM7	−7.24	−7.87	−9.02	−9.15	−9.07	−8.58	−10.05	−10.44	−9.94	−9.66	−9.06	−11.18	−11.02	−8.96	−9.20	−9.62
19	SM8	−9.83	−10.01	−10.58	−11.07	−10.48	−8.28	−11.05	−10.87	−10.64	−10.88	−10.62	−13.19	−12.39	−9.48	−9.33	−10.88
20	SM9	−11.68	−11.38	−11.64	−13.31	−11.88	−11.57	−12.19	−11.98	−11.97	−12.37	−12.09	−14.51	−13.48	−11.25	−9.79	−12.47
21	SM10	−12.08	−11.73	−12.26	−11.83	−12.07	−10.80	−12.88	−13.01	−12.32	−12.33	−11.80	−14.50	−13.72	−10.64	−10.76	−12.24
22	WF1	−7.40	−8.79	−9.30	−9.32	−8.76	−8.49	−11.11	−9.87	−9.31	−9.60	−8.04	−10.97	−10.42	−7.84	−6.94	−8.29
23	WF2	−7.35	−8.45	−8.53	−9.84	−8.11	−6.45	−10.39	−8.96	−8.66	−8.79	−8.02	−10.54	−10.35	−8.12	−7.81	−8.62
24	WF3	−8.42	−9.32	−10.80	−12.11	−9.96	−9.72	−12.26	−11.93	−9.97	−10.80	−9.63	−13.85	−12.05	−8.92	−8.97	−9.93
25	WF4	−8.98	−8.01	−8.89	−14.12	−9.78	−8.56	−11.32	−11.31	−9.68	−10.85	−9.13	−12.39	−10.88	−8.01	−8.45	−10.00
26	WF5	−8.43	−9.75	−9.51	−13.62	−10.34	−9.66	−11.03	−11.25	−11.85	−12.05	−9.52	−13.64	−12.19	−9.70	−10.31	−10.75
27	WF6	−10.03	−12.61	−12.14	−14.40	−11.83	−11.08	−11.73	−13.53	−13.24	−12.21	−11.22	−15.19	−13.84	−10.08	−10.91	−12.44
28	WF7	−10.17	−12.98	−11.41	−14.10	−11.98	−10.63	−12.28	−13.70	−13.66	−12.39	−12.05	−15.09	−12.77	−10.72	−11.93	−12.82
29	WF8	−7.85	−8.44	−8.67	−10.11	−9.28	−8.09		−10.15	−9.39	−9.26	−8.78	−11.59	−10.91	−8.99	−8.99	−9.60
30	BM1	−12.53	−12.87	−12.74	−11.31	−12.38	−10.83	−12.76	−12.86	−12.59	−12.68	−12.37	−12.98	−13.09	−12.45	−12.29	−12.32
31	BM2	−12.16	−12.48	−12.29	−11.37	−11.97	−10.44	−12.31	−12.50	−12.31	−12.35	−12.16	−12.18	−12.33	−12.17	−12.02	−12.09
32	BM3	−14.28	−15.48	−14.40	−15.72	−14.70	−12.89	−15.18	−16.03	−14.58	−15.03	−13.88	−15.92	−15.31	−13.47	−13.32	−13.95
33	BM4	−12.49	−13.07	−11.30	−14.80	−13.38	−10.87	−13.67	−14.77	−13.63	−13.25	−12.03	−14.46	−13.67	−11.52	−11.81	−11.58
34	BM5	−13.01	−14.23	−12.28	−15.66	−13.88	−11.24	−14.24	−15.01	−13.19	−13.63	−12.63	−15.05	−13.68	−11.01	−13.33	−12.76
35	BM6	−15.65	−15.66	−14.54	−16.47	−15.14	−14.17	−15.21	−16.09	−16.00	−14.73	−14.60	−16.92	−15.62	−14.00	−14.89	−14.25
36	BM7	−14.70	−14.44	−15.15	−14.72	−13.01	−12.64	−13.65	−15.00	−14.18	−12.73	−11.79	−15.21	−14.33	−12.31	−12.46	−11.97
37	BM8	−15.15	−15.54	−14.41	−16.54	−15.16	−13.00	−14.51	−16.14	−14.79	−15.81	−14.83	−16.49	−15.07	−13.68	−15.67	−14.16
38	BM9	−13.24	−14.18	−13.95	−14.06	−14.42	−13.57	−14.02	−14.49	−14.02	−14.31	−13.57	−14.56	−15.04	−14.01	−13.91	−13.72
39	BM10	−9.02	−8.12	−8.62	−10.99	−9.59	−8.81	−10.63	−13.42	−9.53	−9.62	−8.22	−10.39	−10.69	−7.42	−8.14	−7.92
40	BM11	−10.87	−11.71	−12.05	−11.86	−11.62	−10.36	−10.57	−12.20	−11.22	−11.24	−7.83	−12.73	−12.95	−15.02	−10.02	−13.84
41	CM1	−15.97	−16.39	−16.10	−16.77	−17.08	−17.51	−17.85	−17.56	−17.32	−17.22	−16.93	−17.52		−16.51	−16.49	−15.98
42	CM2	−13.48	−13.30	−12.86	−14.30	−13.98	−13.58	−13.93	−14.72	−14.19	−13.82	−13.20	−13.90		−13.42	−13.88	−13.55
43	CM3	−15.16	−14.31	−13.70	−16.39	−15.82	−15.72	−15.16	−16.55	−15.82	−15.69	−14.69	−16.67		−14.90	−15.11	−14.99
44	CM4	−16.34	−16.35	−15.83	−15.83	−15.83	−16.72	−17.11	−17.71	−17.03	−17.08	−16.29	−16.80		−16.66	−16.47	−16.69
45	CM5	−12.67	−12.02	−11.32	−15.62	−15.62	−11.90	−12.08	−14.10	−13.91	−12.44	−11.42	−12.60		−11.76	−13.40	−11.44
46	S1	−12.33	−12.49	−12.09	−12.07	−12.19	−11.61	−12.75	−12.49	−12.17	−12.15	−12.10	−12.12	−12.39	−11.90	−12.17	−11.88
47	S2	−15.57	−15.80	−15.82	−16.45	−15.31		−16.04	−16.59	−15.20	−14.65	−14.48	−15.98	−16.24	−14.95	−14.99	−14.98
48	S3	−14.51	−15.23	−15.13	−15.13	−15.13	−15.07	−15.24	−15.82	−14.67	−14.15	−13.87	−14.90	−15.46	−14.13	−14.30	−14.43
49	S4	−13.12	−13.79	−13.59	−15.65	−14.50	−14.66	−14.88	−14.81	−13.89	−13.30	−12.72	−13.79	−14.49	−13.44	−12.94	−13.23
50	S5	−12.46	−12.48	−12.71	−12.71	−12.71		−12.50	−13.37	−11.49	−10.44	−10.37	−12.39	−13.49	−11.03	−11.02	−11.07

**Table 9:** Annual δ^8^H measurements as measured in late summer/early fall for RAMP lake water, northeastern Alberta.

Lake No.	Lake ID	δ^2^H_L_ (per mil)															
		2002	2003	2004	2005	2006	2007	2008	2009	2010	2011	2012	2013	2014	2015	2016	2017
1	NE1	−130.38	−125.70	−126.17	−132.95	−126.31	−119.49	−129.57	−128.76	−116.67	−111.32	−121.97	−131.99	−128.33	−120.06	−125.80	−121.51
2	NE2	−137.66	−123.53	−127.72	−133.13	−133.13	−121.77	−127.41	−129.42	−124.25	−116.19	−123.26	−127.66	−130.00	−116.68	−124.75	−119.48
3	NE3	−117.11	−113.44	−118.30	−126.47	−126.47	−107.61	−114.58	−116.68	−112.27	−107.05	−110.38	−123.18	−121.73	−110.35	−111.16	−112.33
4	NE4	−128.12	−125.93	−128.35	−132.04	−123.23	−115.09	−123.24	−128.67	−119.96	−123.05	−119.16	−128.42	−125.48	−122.63	−124.02	−120.21
5	NE5	−108.48	−107.22	−108.16	−109.55	−103.57	−94.83	−102.68	−111.19	−106.68	−100.43	−102.21	−115.17	−112.63	−103.15	−103.84	−104.40
6	NE6	−127.88	−122.96	−121.17	−131.52	−119.18	−122.70	−102.72	−124.67	−130.00	−122.20	−123.09	−121.52	−123.73	−118.52	−122.47	−123.61
7	NE7	−136.13	−129.44	−129.93	−135.36	−127.37	−127.99	−130.71	−131.21	−131.35	−121.73	−132.75	−134.60	−128.97	−121.49	−129.12	−126.99
8	NE8	−131.89	−124.57	−121.53	−130.97	−122.72	−118.48	−125.83	−126.61	−127.72	−117.31	−123.47	−129.33	−126.19	−112.70	−122.49	−116.54
9	NE9	−94.07	−93.89	−100.72	−101.68	−99.63	−94.17	−99.54	−105.44	−100.63	−95.86	−95.98	−106.16	−104.12	−96.12	−94.90	−95.52
10	NE10	−93.49	−91.77	−101.44	−105.36	−101.46	−96.33	−97.36	−105.58	−96.98	−92.69	−88.47	−103.09	−100.99	−92.36	−91.22	−94.12
11	NE11		−115.98	−114.89	−120.74	−110.98	−101.54	−109.26	−113.40	−106.28	−101.67	−97.72	−121.20	−119.17	−105.27	−105.60	−104.50
12	SM1	−91.22	−89.87	−96.62	−99.32	−91.50	−88.77	−97.30	−101.44	−97.89	−95.28	−86.06	−101.63	−100.47	−91.74	−88.09	−100.22
13	SM2	−87.34	−81.56	−95.07	−101.11	−92.47	−88.65	−93.97	−99.37	−97.68	−95.71	−90.56	−102.66	−98.97	−89.06	−90.32	−96.01
14	SM3	−99.28	−99.01	−100.60	−107.94	−100.59	−99.10	−102.49	−105.88	−106.38	−101.35	−95.97	−107.99	−108.33	−98.86	−93.75	−99.79
15	SM4	−101.32	−106.21	−106.32	−110.70	−106.79	−106.07	−109.60	−113.62	−111.32	−110.13	−105.40	−121.05	−119.00	−99.00	−104.94	−115.32
16	SM5	−99.99	−97.56	−99.68	−102.34	−99.73	−97.99	−104.63	−106.73	−105.87	−100.61	−92.00	−108.48	−107.12	−96.36	−92.72	−98.58
17	SM6	−101.77	−102.78	−105.39	−110.13	−104.45	−103.00	−104.32	−108.89	−106.65	−103.83	−100.43	−117.35	−114.08	−103.23	−95.65	−106.11
18	SM7	−89.34	−89.86	−97.82	−98.18	−95.48	−95.07	−98.74	−102.62	−100.90	−99.21	−93.94	−106.15	−103.72	−95.82	−94.66	−97.18
19	SM8	−100.04	−101.38	−105.15	−108.20	−101.32	−95.58	−101.70	−105.49	−104.40	−102.35	−98.70	−116.23	−111.37	−99.38	−94.64	−103.57
20	SM9	−109.43	−105.15	−108.49	−119.89	−107.76	−109.10	−110.65	−112.90	−112.17	−109.83	−106.58	−123.53	−118.25	−108.09	−98.86	−111.78
21	SM10	−112.35	−108.33	−113.82	−112.19	−108.65	−105.03	−110.93	−117.48	−113.59	−109.92	−106.31	−123.26	−118.99	−106.43	−103.09	−111.29
22	WF1	−89.94	−97.19	−100.54	−99.09	−96.55	−95.81	−101.30	−102.06	−99.75	−97.38	−88.46	−107.70	−103.90	−88.77	−85.87	−92.71
23	WF2	−87.85	−94.29	−96.73	−101.77	−95.54	−87.74	−100.57	−97.75	−95.61	−95.14	−88.91	−105.34	−102.62	−91.05	−89.71	−94.23
24	WF3	−92.71	−98.16	−105.52	−113.61	−101.16	−102.73	−110.39	−112.40	−101.20	−103.22	−95.50	−121.57	−111.85	−94.41	−93.11	−99.24
25	WF4	−105.18	−96.93	−105.09	−124.08	−104.29	−99.44	−111.73	−117.33	−105.87	−111.05	−102.74	−119.14	−113.36	−98.75	−101.30	−107.56
26	WF5	−99.14	−103.85	−103.22	−121.50	−105.21	−102.44	−106.90	−113.43	−111.83	−112.94	−99.58	−121.87	−115.93	−101.58	−104.82	−106.81
27	WF6	−104.75	−113.46	−116.73	−125.58	−111.77	−110.33	−114.99	−124.69	−118.88	−115.29	−107.99	−128.20	−123.02	−105.26	−107.38	−114.70
28	WF7	−106.69	−109.99	−114.94	−124.01	−113.74	−108.82	−116.34	−125.47	−121.46	−114.73	−112.58	−127.93	−118.93	−108.74	−113.25	−115.90
29	WF8	−93.91	−91.58	−94.69	−103.22	−100.31	−95.25		−104.28	−99.32	−97.24	−94.13	−110.10	−107.33	−96.20	−97.02	−99.26
30	BM1	−114.19	−116.64	−115.71	−109.44	−112.38	−108.68	−114.36	−115.68	−114.59	−114.21	−112.83	−116.53	−116.43	−114.44	−112.77	−112.97
31	BM2	−114.60	−112.84	−113.01	−109.76	−111.31	−106.85	−112.61	−112.92	−112.45	−112.93	−111.77	−112.63	−112.99	−112.35	−111.20	−110.83
32	BM3	−124.92	−133.15	−130.03	−132.43	−123.57	−118.20	−130.15	−133.31	−126.90	−130.61	−123.60	−133.32	−133.07	−125.27	−122.38	−123.15
33	BM4	−112.12	−122.22	−112.75	−127.64	−115.26	−108.85	−122.49	−128.06	−120.51	−120.62	−113.00	−126.13	−122.37	−114.44	−112.84	−110.62
34	BM5	−117.85	−125.70	−117.16	−132.10	−119.79	−110.54	−125.02	−128.80	−117.71	−122.31	−114.15	−127.42	−122.49	−110.55	−120.26	−116.42
35	BM6	−131.00	−135.88	−129.37	−136.36	−124.41	−124.16	−130.53	−135.92	−131.49	−127.61	−126.02	−140.30	−132.59	−125.72	−129.18	−125.55
36	BM7	−125.55	−125.43	−130.52	−127.20	−118.52	−116.76	−120.68	−129.78	−122.21	−115.68	−109.73	−127.95	−123.47	−116.22	−115.21	−114.09
37	BM8	−128.36	−133.40	−128.60	−136.70	−125.43	−118.74	−131.74	−134.09	−124.83	−132.13	−125.99	−132.49	−129.08	−124.53	−130.33	−123.19
38	BM9	−116.66	−124.75	−123.14	−123.77	−122.93	−121.38	−123.58	−125.53	−122.69	−123.65	−120.26	−124.75	−126.33	−123.71	−121.97	−120.86
39	BM10	−101.85	−96.32	−98.49	−107.79	−103.53	−96.57	−103.61	−121.72	−101.70	−100.07	−92.81	−104.91	−106.10	−92.80	−94.61	−93.71
40	BM11	−110.11	−114.18	−114.80	−112.30	−111.97	−106.28	−111.13	−117.20	−110.44	−109.61	−91.18	−117.69	−119.98	−130.10	−106.73	−123.28
41	CM1	−134.34	−134.20	−136.70	−137.90	−138.64	−142.19	−142.85	−143.28	−141.01	−141.79	−139.46	−143.24		−138.44	−136.06	−134.72
42	CM2	−120.92	−120.17	−118.16	−125.04	−124.11	−123.57	−123.53	−128.62	−125.90	−124.07	−120.75	−124.11		−121.91	−122.85	−121.23
43	CM3	−130.92	−128.42	−125.31	−135.94	−134.37	−133.68	−131.07	−139.63	−136.98	−135.16	−127.53	−138.14		−130.30	−130.09	−129.57
44	CM4	−138.09	−136.28	−135.94	−135.94	−135.94	−138.42	−141.45	−145.10	−140.66	−141.81	−136.73	−139.76		−138.78	−137.26	−137.79
45	CM5	−116.86	−115.14	−112.39	−131.93	−131.93	−115.58	−115.12	−127.87	−124.40	−118.52	−110.04	−116.59		−113.88	−121.33	−113.74
46	S1	−115.02	−116.15	−116.52	−113.40	−116.08	−115.11	−115.56	−116.23	−114.33	−113.36	−112.59	−114.29	−114.18	−113.34	−113.57	−113.61
47	S2	−132.07	−134.77	−136.63	−136.22	−132.57		−137.39	−136.97	−130.65	−127.42	−127.09	−133.15	−134.19	−129.81	−129.26	−130.20
48	S3	−127.44	−131.88	−132.34	−132.34	−132.34	−131.07	−133.43	−133.50	−129.26	−124.72	−123.39	−128.93	−130.03	−125.89	−126.03	−126.74
49	S4	−120.66	−125.65	−125.65	−132.06	−128.90	−129.20	−131.23	−130.09	−125.37	−121.80	−119.01	−124.29	−127.32	−123.12	−119.90	−121.71
50	S5	−116.70	−121.84	−123.13	−123.13	−123.13		−118.70	−121.85	−111.99	−106.48	−105.86	−115.50	−120.23	−111.47	−109.83	−111.08

**Table 10:** Mean annual stable isotope data for RAMP sites, northeastern Alberta.

Lake No.	Lake ID	δ^18^O (‰)	δ^2^H (‰)	δ^18^O_L_ (‰)	δ^2^H_L_ (‰)	δ^18^O_A_ (‰)	δ^2^H_A_ (‰)
		Precipitation		Lake water		Atmospheric moisture	
1	NE1	−18.34	−142.37	−14.65	−124.81	−22.48	−173.35
2	NE2	−18.33	−142.31	−14.87	−126.00	−21.56	−166.01
3	NE3	−17.82	−138.62	−12.84	−115.57	−21.72	−167.17
4	NE4	−18.79	−145.57	−13.96	−124.22	−22.01	−169.55
5	NE5	−18.03	−140.11	−10.82	−105.89	−21.69	−166.92
6	NE6	−18.35	−142.44	−14.05	−122.37	−21.49	−165.49
7	NE7	−18.34	−142.36	−15.69	−129.70	−21.66	−167.04
8	NE8	−18.35	−142.44	−14.33	−123.65	−21.70	−167.03
9	NE9	−17.97	−139.67	−9.56	−98.65	−23.23	−179.29
10	NE10	−17.92	−139.33	−9.27	−97.04	−23.60	−183.19
11	NE11	−17.89	−139.09	−11.39	−109.88	−21.72	−167.15
12	SM1	−17.77	−138.21	−9.04	−94.84	−21.71	−167.11
13	SM2	−17.98	−139.77	−8.80	−93.78	−21.98	−169.66
14	SM3	−18.24	−141.59	−10.15	−101.71	−21.97	−169.58
15	SM4	−18.22	−141.50	−11.84	−109.17	−21.95	−169.42
16	SM5	−18.22	−141.47	−10.00	−100.65	−21.98	−169.65
17	SM6	−18.25	−141.68	−11.09	−105.50	−21.62	−166.54
18	SM7	−17.93	−139.41	−9.38	−97.42	−21.95	−169.43
19	SM8	−18.24	−141.66	−10.60	−103.09	−21.98	−169.68
20	SM9	−18.24	−141.66	−12.10	−110.78	−21.47	−165.36
21	SM10	−18.27	−141.83	−12.19	−111.35	−21.71	−167.46
22	WF1	−18.07	−140.41	−9.03	−96.69	−23.63	−183.37
23	WF2	−18.06	−140.36	−8.69	−95.30	−22.18	−170.93
24	WF3	−17.80	−138.46	−10.54	−103.55	−21.77	−167.79
25	WF4	−17.88	−139.04	−10.02	−107.74	−21.77	−167.74
26	WF5	−18.05	−140.24	−10.85	−108.19	−22.01	−169.84
27	WF6	−18.03	−140.08	−12.28	−115.19	−22.03	−169.66
28	WF7	−18.05	−140.29	−12.42	−115.84	−22.50	−173.97
29	WF8	−18.04	−140.19	−8.76	−92.74	−22.44	−173.44
30	BM1	−18.84	−145.93	−12.44	−113.87	−22.51	−174.01
31	BM2	−18.71	−145.02	−12.07	−111.94	−21.65	−166.66
32	BM3	−18.73	−145.17	−14.63	−127.75	−21.52	−165.77
33	BM4	−18.69	−144.86	−12.89	−118.12	−23.50	−182.23
34	BM5	−18.76	−145.37	−13.43	−120.52	−21.56	−165.88
35	BM6	−18.89	−146.33	−15.25	−130.38	−22.79	−176.22
36	BM7	−19.04	−147.37	−13.64	−121.19	−23.58	−182.74
37	BM8	−18.73	−145.15	−15.06	−128.73	−22.64	−175.11
38	BM9	−18.87	−146.17	−14.07	−122.87	−22.57	−174.39
39	BM10	−18.40	−142.78	−9.45	−101.04	−22.73	−175.80
40	BM11	−18.96	−146.79	−11.63	−112.94	−22.12	−170.66
41	CM1	−19.55	−151.05	−15.82	−138.99	−22.59	−174.35
42	CM2	−19.76	−152.60	−12.88	−122.99	−22.12	−170.46
43	CM3	−19.79	−152.79	−14.42	−132.47	−22.43	−173.08
44	CM4	−19.80	−152.88	−15.55	−138.66	−22.54	−173.94
45	CM5	−19.69	−152.10	−12.02	−119.02	−22.02	−169.65
46	S1	−18.84	−145.93	−12.18	−114.58	−22.01	−169.54
47	S2	−18.45	−143.14	−15.54	−132.56	−22.01	−169.48
48	S3	−18.56	−143.93	−14.82	−129.33	−22.24	−171.39
49	S4	−18.50	−143.51	−13.93	−125.37	−22.49	−173.78
50	S5	−18.60	−144.21	−12.02	−116.06	−22.27	−171.65

**Table 11:** Site-specific evaporation/inflow index ratios for RAMP Lakes, northeastern Alberta.

Lake No.	Lake ID	E/I															
		2002	2003	2004	2005	2006	2007	2008	2009	2010	2011	2012	2013	2014	2015	2016	2017
1	NE1	0.069	0.101	0.129	0.076	0.114	0.182	0.057	0.095	0.195	0.275	0.138	0.066	0.104	0.184	0.110	0.151
2	NE2	0.051	0.099	0.123	0.074	0.074	0.157	0.082	0.086	0.117	0.223	0.119	0.088	0.084	0.236	0.103	0.166
3	NE3	0.175	0.167	0.173	0.102	0.103	0.306	0.161	0.161	0.206	0.267	0.226	0.108	0.131	0.261	0.213	0.209
4	NE4	0.127	0.165	0.165	0.102	0.203	0.263	0.154	0.124	0.219	0.198	0.226	0.141	0.160	0.227	0.180	0.226
5	NE5	0.312	0.272	0.298	0.281	0.390	0.631	0.312	0.256	0.313	0.395	0.355	0.189	0.233	0.357	0.330	0.332
6	NE6	0.102	0.143	0.192	0.086	0.205	0.111	0.363	0.134	0.081	0.168	0.133	0.143	0.138	0.198	0.145	0.144
7	NE7	0.040	0.077	0.085	0.060	0.080	0.074	0.060	0.076	0.069	0.164	0.057	0.050	0.104	0.179	0.073	0.107
8	NE8	0.076	0.128	0.168	0.090	0.164	0.192	0.081	0.110	0.093	0.202	0.122	0.089	0.136	0.282	0.138	0.200
9	NE9	0.432	0.461	0.416	0.397	0.419	0.660	0.435	0.305	0.380	0.455	0.463	0.292	0.317	0.461	0.474	0.471
10	NE10	0.462	0.564	0.391	0.331	0.437	0.485	0.421	0.297	0.430	0.493	0.580	0.341	0.345	0.533	0.582	0.479
11	NE11		0.200	0.211	0.152	0.287	0.464	0.248	0.221	0.302	0.374	0.432	0.150	0.147	0.354	0.342	0.343
12	SM1	0.476	0.520	0.421	0.421	0.560	0.744	0.352	0.349	0.390	0.420	0.583	0.337	0.365	0.476	0.576	0.350
13	SM2	0.642	0.729	0.481	0.404	0.546	0.795	0.407	0.396	0.422	0.435	0.496	0.324	0.398	0.580	0.580	0.448
14	SM3	0.407	0.422	0.407	0.314	0.401	0.463	0.355	0.318	0.341	0.383	0.451	0.269	0.274	0.440	0.525	0.440
15	SM4	0.375	0.265	0.293	0.275	0.292	0.314	0.242	0.221	0.241	0.236	0.289	0.120	0.159	0.429	0.279	0.177
16	SM5	0.385	0.461	0.422	0.406	0.454	0.492	0.289	0.315	0.316	0.393	0.526	0.259	0.271	0.458	0.564	0.454
17	SM6	0.352	0.382	0.321	0.284	0.328	0.374	0.282	0.285	0.309	0.297	0.330	0.161	0.207	0.375	0.463	0.285
18	SM7	0.623	0.587	0.460	0.454	0.470	0.537	0.366	0.329	0.372	0.396	0.455	0.283	0.294	0.444	0.461	0.414
19	SM8	0.390	0.396	0.347	0.311	0.364	0.631	0.310	0.319	0.342	0.323	0.347	0.179	0.225	0.422	0.477	0.328
20	SM9	0.262	0.290	0.269	0.173	0.257	0.275	0.236	0.247	0.253	0.229	0.246	0.118	0.167	0.296	0.428	0.222
21	SM10	0.240	0.267	0.231	0.259	0.246	0.336	0.199	0.192	0.233	0.232	0.266	0.119	0.156	0.338	0.342	0.237
22	WF1	0.649	0.501	0.452	0.455	0.534	0.516	0.298	0.384	0.437	0.418	0.596	0.312	0.353	0.562	0.826	0.581
23	WF2	0.655	0.540	0.539	0.402	0.627	1.047	0.352	0.463	0.500	0.498	0.598	0.346	0.358	0.533	0.662	0.537
24	WF3	0.492	0.420	0.299	0.216	0.375	0.397	0.209	0.229	0.360	0.302	0.396	0.128	0.222	0.437	0.474	0.375
25	WF4	0.440	0.577	0.474	0.123	0.400	0.536	0.271	0.270	0.391	0.303	0.455	0.205	0.304	0.533	0.545	0.376
26	WF5	0.508	0.407	0.429	0.151	0.364	0.430	0.305	0.287	0.252	0.240	0.440	0.149	0.230	0.401	0.370	0.331
27	WF6	0.364	0.205	0.228	0.116	0.251	0.301	0.256	0.159	0.175	0.229	0.294	0.085	0.142	0.370	0.319	0.215
28	WF7	0.356	0.187	0.276	0.130	0.243	0.339	0.225	0.153	0.156	0.220	0.241	0.089	0.197	0.325	0.249	0.196
29	WF8	0.572	0.543	0.519	0.377	0.471	0.642		0.366	0.437	0.452	0.521	0.266	0.316	0.462	0.507	0.435
30	BM1	0.248	0.234	0.239	0.339	0.265	0.384	0.237	0.231	0.250	0.244	0.266	0.222	0.220	0.260	0.270	0.267
31	BM2	0.263	0.249	0.259	0.324	0.284	0.409	0.259	0.247	0.262	0.259	0.273	0.268	0.260	0.271	0.283	0.277
32	BM3	0.152	0.103	0.146	0.091	0.133	0.221	0.114	0.083	0.143	0.122	0.175	0.083	0.110	0.198	0.200	0.170
33	BM4	0.241	0.211	0.321	0.125	0.195	0.361	0.179	0.130	0.185	0.204	0.277	0.140	0.181	0.307	0.293	0.308
34	BM5	0.216	0.157	0.260	0.094	0.173	0.337	0.156	0.123	0.212	0.188	0.244	0.118	0.184	0.347	0.201	0.235
35	BM6	0.102	0.103	0.148	0.070	0.121	0.161	0.122	0.088	0.095	0.146	0.154	0.057	0.109	0.185	0.136	0.169
36	BM7	0.148	0.162	0.129	0.146	0.238	0.257	0.199	0.134	0.176	0.251	0.316	0.123	0.166	0.278	0.271	0.304
37	BM8	0.115	0.100	0.145	0.062	0.114	0.213	0.143	0.079	0.134	0.092	0.132	0.064	0.120	0.187	0.093	0.159
38	BM9	0.209	0.163	0.173	0.167	0.152	0.190	0.172	0.149	0.176	0.161	0.198	0.144	0.127	0.178	0.176	0.188
39	BM10	0.482	0.620	0.550	0.328	0.463	0.559	0.358	0.181	0.447	0.445	0.624	0.388	0.356	0.660	0.666	0.690
40	BM11	0.365	0.312	0.285	0.302	0.324	0.431	0.401	0.275	0.347	0.345	0.726	0.245	0.235	0.137	0.482	0.186
41	CM1	0.115	0.100	0.115	0.084	0.072	0.057	0.048	0.060	0.068	0.072	0.085	0.058		0.107	0.099	0.123
42	CM2	0.241	0.255	0.281	0.198	0.217	0.236	0.218	0.175	0.207	0.226	0.265	0.218		0.252	0.223	0.243
43	CM3	0.157	0.200	0.235	0.106	0.129	0.131	0.161	0.101	0.133	0.140	0.191	0.096		0.180	0.164	0.171
44	CM4	0.108	0.108	0.134	0.129	0.129	0.092	0.080	0.059	0.084	0.083	0.116	0.088		0.102	0.102	0.096
45	CM5	0.287	0.339	0.384	0.133	0.134	0.351	0.336	0.206	0.221	0.310	0.385	0.300		0.356	0.250	0.387
46	S1	0.262	0.258	0.278	0.283	0.280	0.313	0.244	0.256	0.281	0.280	0.286	0.281	0.266	0.296	0.280	0.298
47	S2	0.090	0.083	0.084	0.061	0.104		0.078	0.056	0.111	0.132	0.141	0.077	0.072	0.123	0.115	0.120
48	S3	0.136	0.109	0.114	0.114	0.116	0.116	0.113	0.087	0.138	0.159	0.174	0.123	0.105	0.164	0.149	0.148
49	S4	0.199	0.169	0.179	0.091	0.140	0.131	0.125	0.125	0.171	0.198	0.231	0.170	0.143	0.193	0.215	0.203
50	S5	0.242	0.246	0.230	0.232	0.235		0.245	0.196	0.310	0.386	0.395	0.251	0.194	0.337	0.349	0.339

**Table 12:** Site-specific annual water yield to lakes, RAMP sites, northeastern Alberta.

Lake No.	Lake ID	Wy(mm)															
		2002	2003	2004	2005	2006	2007	2008	2009	2010	2011	2012	2013	2014	2015	2016	2017
1	NE1	197	194	133	265	180	98	369	208	91	62	145	334	202	106	189	130
2	NE2	153	111	79	152	161	66	140	130	94	45	97	137	144	44	115	65
3	NE3	88	132	112	232	248	58	145	143	109	80	98	251	197	86	106	113
4	NE4	606	503	449	869	409	260	603	717	368	433	355	673	572	380	440	359
5	NE5	267	488	379	480	303	101	433	577	442	329	345	950	732	412	409	427
6	NE6	156	148	91	260	101	192	42	161	289	131	175	163	170	113	158	158
7	NE7	166	125	101	162	126	132	165	125	143	56	188	215	97	52	141	91
8	NE8	753	586	373	861	461	349	1007	704	867	364	674	972	601	253	582	373
9	NE9	176	245	255	339	319	106	273	507	367	294	223	604	547	320	242	273
10	NE10	132	128	230	373	246	189	247	432	243	209	113	402	404	209	132	228
11	NE11		167	140	239	112	47	129	151	100	79	48	266	273	93	86	90
12	SM1	132	181	230	277	143	49	399	389	315	288	152	407	370	235	127	386
13	SM2	31	33	72	126	65	10	131	143	112	116	93	184	137	80	46	111
14	SM3	182	260	236	433	296	211	359	428	369	327	231	547	532	275	150	267
15	SM4	29	73	57	72	69	58	88	97	86	91	72	210	150	40	67	132
16	SM5	241	258	260	347	274	218	592	525	501	380	198	696	654	312	151	306
17	SM6	39	51	60	84	69	53	85	84	72	79	68	177	129	58	34	87
18	SM7	56	117	142	193	171	116	285	341	254	249	200	413	394	230	156	235
19	SM8	144	213	230	323	256	70	325	313	271	309	274	685	509	215	138	314
20	SM9	156	205	204	412	259	225	287	265	251	294	275	670	440	211	103	320
21	SM10	95	124	136	135	149	90	193	196	151	156	134	358	257	96	83	159
22	WF1	98	235	252	305	218	200	631	455	321	361	185	593	526	244	−8	210
23	WF2	46	96	81	182	69	−25	236	165	121	125	88	242	248	129	33	119
24	WF3	19	35	51	91	43	34	103	89	44	62	42	185	100	37	22	46
25	WF4	9	8	10	78	17	9	29	29	16	26	12	46	27	13	9	20
26	WF5	14	38	30	156	49	34	64	70	83	94	34	175	104	52	47	62
27	WF6	27	99	77	196	81	61	75	134	123	94	63	309	173	53	55	102
28	WF7	34	138	73	214	105	62	114	177	176	121	107	363	146	79	100	143
29	WF8	20	42	38	93	61	25		98	70	74	45	168	135	83	51	81
30	BM1	431	660	595	435	607	343	711	707	631	673	623	800	810	670	567	629
31	BM2	353	536	472	410	487	263	571	590	532	575	551	576	606	584	488	575
32	BM3	77	141	87	168	112	59	135	183	97	120	83	193	143	73	68	91
33	BM4	167	232	119	455	274	112	305	426	272	254	174	417	311	164	154	166
34	BM5	141	244	118	455	232	92	264	332	169	205	151	371	223	102	191	172
35	BM6	393	455	285	733	407	284	444	608	565	354	351	998	496	287	374	319
36	BM7	430	444	531	514	287	245	337	504	350	241	185	635	445	233	224	229
37	BM8	121	168	101	289	151	69	115	212	112	178	132	297	147	87	199	116
38	BM9	179	288	246	295	326	239	278	309	243	286	243	365	415	279	284	288
39	BM10	30	25	27	92	51	33	81	199	53	59	24	77	90	35	17	29
40	BM11	75	117	121	133	116	69	77	128	86	97	14	174	183	344	53	266
41	CM1	240	310	235	378	455	551	873	648	575	559	460	691		341	392	311
42	CM2	304	328	234	447	404	328	401	495	449	385	290	427		292	402	378
43	CM3	189	162	111	331	275	249	241	387	314	289	202	431		217	228	238
44	CM4	242	275	182	219	228	308	389	503	377	367	270	361		300	308	342
45	CM5	225	212	136	697	704	175	221	401	409	258	169	282		174	347	187
46	S1	425	482	387	389	452	349	509	455	419	447	460	472	471	399	401	450
47	S2	43	51	42	65	39		53	70	34	31	30	57	54	28	34	33
48	S3	112	159	130	140	148	139	153	191	116	110	100	148	156	87	109	112
49	S4	23	30	24	57	38	38	43	40	29	27	22	32	35	22	22	25
50	S5	113	122	108	116	127		118	145	84	70	65	122	149	64	67	78

Mann-Kendall test data (including tau values, p values, trends, n values; Table 13, Table 14) were calculated using the statistical program R to allow for basic assessment of possible parameter trends over the monitoring period. p values less than 0.05 were confirmed to be statistically significant trends.

**Table 13:** Site-specific Mann-Kendall tau and p trend data, RAMP sites, northeastern Alberta.

Lake No.	Lake ID	Mann-Kendall tau values												Mann-Kendall p values											
		δ^18^O_L_ (‰)	δ^2^H_L_ (‰)	T (°C)	T fw (°C)	h	h fw	P(mm)	E(mm)	E/I	Wy(mm)	Wy/P	τ	δ^18^O_L_ (‰)	δ^2^H_L_ (‰)	T (°C)	T fw (°C)	h	h fw	P(mm)	E(mm)	E/I	Wy(mm)	Wy/P	τ
1	NE1	0.25	0.22	0.27	0.25	0.15	0.03	−0.08	0.52	0.25	−0.15	−0.15	0.15	0.19	0.26	0.16	0.19	0.44	0.89	0.69	0.01	0.19	0.44	0.44	0.44
2	NE2	0.38	0.34	0.27	0.25	0.15	0.03	−0.08	0.52	0.38	−0.28	−0.25	0.28	0.05	0.07	0.16	0.19	0.44	0.89	0.69	0.01	0.04	0.14	0.19	0.14
3	NE3	0.18	0.23	0.28	0.28	0.20	0.12	−0.07	0.62	0.17	−0.02	−0.12	0.02	0.37	0.24	0.14	0.14	0.30	0.56	0.75	0.00	0.39	0.96	0.56	0.96
4	NE4	0.30	0.25	0.22	0.22	0.03	−0.20	−0.10	0.38	0.28	−0.25	−0.17	0.25	0.12	0.19	0.26	0.26	0.89	0.30	0.62	0.04	0.14	0.19	0.39	0.19
5	NE5	0.03	0.05	0.32	0.33	0.22	0.10	0.08	0.58	0.08	0.20	0.12	−0.20	0.89	0.82	0.10	0.08	0.26	0.62	0.69	0.00	0.69	0.30	0.56	0.30
6	NE6	0.12	0.10	0.30	0.32	0.23	0.12	0.03	0.65	0.17	0.07	0.02	−0.07	0.56	0.62	0.12	0.10	0.22	0.56	0.89	0.00	0.39	0.75	0.96	0.75
7	NE7	0.17	0.23	0.27	0.25	0.15	0.03	−0.08	0.52	0.18	−0.17	−0.23	0.17	0.39	0.22	0.16	0.19	0.44	0.89	0.69	0.01	0.34	0.39	0.22	0.39
8	NE8	0.30	0.28	0.30	0.32	0.23	0.12	0.03	0.65	0.30	−0.17	−0.18	0.17	0.12	0.14	0.12	0.10	0.22	0.56	0.89	0.00	0.12	0.39	0.34	0.39
9	NE9	−0.08	−0.07	0.30	0.30	0.20	0.02	0.07	0.50	0.12	0.22	0.18	−0.22	0.69	0.75	0.12	0.12	0.30	0.96	0.75	0.01	0.56	0.26	0.34	0.26
10	NE10	0.13	0.18	0.28	0.30	0.07	0.03	−0.02	0.47	0.17	0.05	0.18	−0.05	0.50	0.34	0.14	0.12	0.75	0.89	0.96	0.01	0.39	0.82	0.34	0.82
11	NE11	0.20	0.30	0.30	0.30	0.22	0.15	0.03	0.65	0.20	−0.20	−0.18	0.20	0.32	0.14	0.12	0.12	0.26	0.44	0.89	0.00	0.32	0.32	0.37	0.32
12	SM1	−0.15	−0.13	0.30	0.30	0.10	−0.02	0.17	0.33	−0.15	0.23	0.18	−0.23	0.44	0.50	0.12	0.12	0.62	0.96	0.39	0.08	0.44	0.22	0.34	0.22
13	SM2	−0.18	−0.18	0.18	0.20	0.22	0.03	0.23	0.48	−0.15	0.23	0.18	−0.23	0.34	0.34	0.34	0.30	0.26	0.89	0.22	0.01	0.44	0.22	0.34	0.22
14	SM3	0.05	−0.02	0.17	0.20	0.07	−0.02	0.07	0.32	0.05	0.10	0.12	−0.10	0.82	0.96	0.39	0.30	0.75	0.96	0.75	0.10	0.82	0.62	0.56	0.62
15	SM4	−0.35	−0.22	0.28	0.25	0.12	0.02	0.02	0.32	−0.33	0.32	0.37	−0.32	0.06	0.26	0.14	0.19	0.56	0.96	0.96	0.10	0.08	0.10	0.05	0.10
16	SM5	0.03	−0.02	0.15	0.18	0.07	−0.02	0.07	0.33	0.05	0.13	0.08	−0.13	0.89	0.96	0.44	0.34	0.75	0.96	0.75	0.08	0.82	0.50	0.69	0.50
17	SM6	−0.17	−0.07	0.18	0.22	0.07	−0.02	0.08	0.32	−0.10	0.27	0.20	−0.27	0.39	0.75	0.34	0.26	0.75	0.96	0.69	0.10	0.62	0.16	0.30	0.16
18	SM7	−0.37	−0.27	0.13	0.13	0.23	0.10	0.23	0.42	−0.37	0.38	0.33	−0.38	0.05	0.16	0.50	0.50	0.22	0.62	0.22	0.03	0.05	0.04	0.08	0.04
19	SM8	−0.17	−0.02	0.18	0.22	0.07	−0.02	0.07	0.32	−0.08	0.23	0.27	−0.23	0.39	0.96	0.34	0.26	0.75	0.96	0.75	0.10	0.69	0.22	0.16	0.22
20	SM9	−0.20	−0.08	0.18	0.22	0.07	−0.02	0.08	0.32	−0.20	0.28	0.23	−0.28	0.30	0.69	0.34	0.26	0.75	0.96	0.69	0.10	0.30	0.14	0.22	0.14
21	SM10	−0.07	0.07	0.18	0.22	0.07	−0.02	0.08	0.32	−0.02	0.22	0.13	−0.22	0.75	0.75	0.34	0.26	0.75	0.96	0.69	0.10	0.96	0.26	0.50	0.26
22	WF1	0.03	0.07	0.27	0.25	0.17	0.02	0.13	0.37	0.02	0.10	0.07	−0.32	0.89	0.75	0.16	0.19	0.39	0.96	0.50	0.05	0.96	0.62	0.75	0.10
23	WF2	−0.10	0.00	0.27	0.27	0.13	−0.05	0.08	0.33	−0.12	0.18	0.20	−0.10	0.62	1.00	0.16	0.16	0.50	0.82	0.69	0.08	0.56	0.34	0.30	0.62
24	WF3	−0.02	0.03	0.28	0.27	0.15	−0.02	0.12	0.38	−0.02	0.12	0.17	−0.12	0.96	0.89	0.14	0.16	0.44	0.96	0.56	0.04	0.96	0.56	0.39	0.56
25	WF4	−0.02	−0.03	0.20	0.23	0.10	0.07	0.02	0.55	−0.02	0.13	0.17	−0.13	0.96	0.89	0.30	0.22	0.62	0.75	0.96	0.00	0.96	0.50	0.39	0.50
26	WF5	−0.28	−0.18	0.25	0.25	0.22	0.07	0.07	0.53	−0.27	0.30	0.27	−0.30	0.14	0.34	0.19	0.19	0.26	0.75	0.75	0.00	0.16	0.12	0.16	0.12
27	WF6	−0.02	0.00	0.25	0.25	0.18	0.07	−0.07	0.40	0.02	0.07	0.12	−0.07	0.96	1.00	0.19	0.19	0.34	0.75	0.75	0.03	0.96	0.75	0.56	0.75
28	WF7	−0.12	−0.13	0.25	0.25	0.18	0.07	−0.12	0.40	−0.12	0.15	0.20	−0.15	0.56	0.50	0.19	0.19	0.34	0.75	0.56	0.03	0.56	0.44	0.30	0.44
29	WF8	−0.33	−0.26	0.25	0.25	0.22	0.07	0.07	0.53	−0.31	0.37	0.35	−0.37	0.09	0.20	0.19	0.19	0.26	0.75	0.75	0.00	0.11	0.06	0.07	0.06
30	BM1	0.05	0.02	0.08	0.10	0.13	0.10	0.08	0.47	0.05	0.28	0.30	−0.28	0.82	0.96	0.69	0.62	0.50	0.62	0.69	0.01	0.82	0.14	0.12	0.14
31	BM2	0.07	0.22	0.25	0.27	0.17	0.08	0.03	0.60	0.18	0.47	0.27	−0.47	0.75	0.26	0.19	0.16	0.39	0.69	0.89	0.00	0.34	0.01	0.16	0.01
32	BM3	0.15	0.12	0.22	0.23	0.13	0.02	0.15	0.60	0.15	−0.07	−0.10	0.07	0.44	0.56	0.26	0.22	0.50	0.96	0.44	0.00	0.44	0.75	0.62	0.75
33	BM4	0.10	0.07	0.22	0.23	0.13	0.00	0.15	0.60	0.12	−0.08	−0.03	0.08	0.62	0.75	0.26	0.22	0.50	1.00	0.44	0.00	0.56	0.69	0.89	0.69
34	BM5	0.13	0.15	0.22	0.23	0.13	0.02	0.15	0.60	0.13	−0.07	−0.08	0.07	0.50	0.44	0.26	0.22	0.50	0.96	0.44	0.00	0.50	0.75	0.69	0.75
35	BM6	0.20	0.17	0.50	0.48	−0.10	−0.30	0.08	0.83	0.25	−0.08	−0.10	0.08	0.30	0.39	0.01	0.01	0.62	0.12	0.69	0.00	0.19	0.69	0.62	0.69
36	BM7	0.38	0.43	0.10	0.10	0.10	0.02	0.08	0.38	0.38	−0.37	−0.32	0.37	0.04	0.02	0.62	0.62	0.62	0.96	0.69	0.04	0.04	0.05	0.10	0.05
37	BM8	0.07	0.18	0.22	0.22	0.08	−0.05	0.12	0.53	0.07	0.03	0.05	−0.03	0.75	0.34	0.26	0.26	0.69	0.82	0.56	0.00	0.75	0.89	0.82	0.89
38	BM9	−0.05	0.00	0.22	0.22	0.08	−0.05	0.12	0.53	−0.03	0.23	0.17	−0.23	0.82	1.00	0.26	0.26	0.69	0.82	0.56	0.00	0.89	0.22	0.39	0.22
39	BM10	0.12	0.18	0.32	0.28	0.20	0.10	0.08	0.63	0.20	0.02	0.00	−0.02	0.56	0.34	0.10	0.14	0.30	0.62	0.69	0.00	0.30	0.96	1.00	0.96
40	BM11	−0.20	−0.15	0.18	0.20	0.08	−0.02	0.05	0.40	−0.20	0.22	0.20	−0.22	0.30	0.44	0.34	0.30	0.69	0.96	0.82	0.03	0.30	0.26	0.30	0.26
41	CM1	−0.07	−0.16	0.72	0.72	0.08	−0.18	0.13	0.72	0.01	0.16	0.18	−0.16	0.77	0.43	0.00	0.00	0.69	0.34	0.50	0.00	1.00	0.43	0.37	0.43
42	CM2	0.01	−0.03	0.23	0.23	0.07	−0.12	0.03	0.23	0.01	0.09	0.14	−0.09	1.00	0.92	0.22	0.22	0.75	0.56	0.89	0.22	1.00	0.69	0.49	0.69
43	CM3	−0.01	0.01	0.42	0.40	0.07	−0.22	0.25	0.70	0.03	0.18	0.12	−0.18	1.00	1.00	0.03	0.03	0.75	0.26	0.19	0.00	0.92	0.37	0.55	0.37
44	CM4	−0.21	−0.19	0.05	0.05	0.12	−0.07	0.03	0.53	−0.24	0.28	0.33	−0.28	0.30	0.34	0.82	0.82	0.56	0.75	0.89	0.00	0.23	0.17	0.09	0.17
45	CM5	0.15	0.19	0.25	0.25	0.00	−0.17	0.13	0.37	0.22	−0.07	−0.10	0.07	0.46	0.35	0.19	0.19	1.00	0.39	0.50	0.05	0.28	0.77	0.62	0.77
46	S1	0.23	0.45	0.22	0.23	−0.03	−0.28	0.13	0.47	0.33	0.08	−0.08	−0.08	0.22	0.02	0.26	0.22	0.89	0.14	0.50	0.01	0.08	0.69	0.69	0.69
47	S2	0.24	0.33	0.35	0.35	−0.12	−0.38	0.02	0.13	0.24	−0.30	−0.28	0.30	0.23	0.09	0.06	0.06	0.56	0.04	0.96	0.50	0.23	0.14	0.17	0.14
48	S3	0.28	0.33	0.38	0.37	−0.10	−0.33	0.03	0.18	0.32	−0.22	−0.27	0.22	0.15	0.09	0.04	0.05	0.62	0.08	0.89	0.34	0.10	0.26	0.16	0.26
49	S4	0.27	0.30	0.38	0.37	−0.10	−0.33	0.03	0.18	0.27	−0.27	−0.27	0.27	0.16	0.12	0.04	0.05	0.62	0.08	0.89	0.34	0.16	0.16	0.16	0.16
50	S5	0.27	0.46	0.35	0.35	−0.12	−0.38	0.02	0.13	0.35	−0.24	−0.39	0.24	0.18	0.02	0.06	0.06	0.56	0.04	0.96	0.50	0.07	0.23	0.05	0.23

**Table 14:** Mann-Kendall trend results and number of years observations.

Lake No.	Lake ID	Mann-Kendall trend/no trend												Mann-Kendall number of years observations											
		δ^18^O_L_ (‰)	δ^2^H_L_ (‰)	T (°C)	T fw (°C)	h	h fw	P(mm)	E(mm)	E/I	Wy(mm)	Wy/P	τ	δ^18^O_L_ (‰)	δ^2^H_L_ (‰)	T (°C)	T fw (°C)	h	h fw	P(mm)	E(mm)	E/I	Wy(mm)	Wy/P	τ
1	NE1	No.Trend	No.Trend	No.Trend	No.Trend	No.Trend	No.Trend	No.Trend	Upward	No.Trend	No.Trend	No.Trend	No.Trend	16	16	16	16	16	16	16	16	16	16	16	16
2	NE2	Upward	No.Trend	No.Trend	No.Trend	No.Trend	No.Trend	No.Trend	Upward	Upward	No.Trend	No.Trend	No.Trend	16	16	16	16	16	16	16	16	16	16	16	16
3	NE3	No.Trend	No.Trend	No.Trend	No.Trend	No.Trend	No.Trend	No.Trend	Upward	No.Trend	No.Trend	No.Trend	No.Trend	16	16	16	16	16	16	16	16	16	16	16	16
4	NE4	No.Trend	No.Trend	No.Trend	No.Trend	No.Trend	No.Trend	No.Trend	Upward	No.Trend	No.Trend	No.Trend	No.Trend	16	16	16	16	16	16	16	16	16	16	16	16
5	NE5	No.Trend	No.Trend	No.Trend	No.Trend	No.Trend	No.Trend	No.Trend	Upward	No.Trend	No.Trend	No.Trend	No.Trend	16	16	16	16	16	16	16	16	16	16	16	16
6	NE6	No.Trend	No.Trend	No.Trend	No.Trend	No.Trend	No.Trend	No.Trend	Upward	No.Trend	No.Trend	No.Trend	No.Trend	16	16	16	16	16	16	16	16	16	16	16	16
7	NE7	No.Trend	No.Trend	No.Trend	No.Trend	No.Trend	No.Trend	No.Trend	Upward	No.Trend	No.Trend	No.Trend	No.Trend	16	16	16	16	16	16	16	16	16	16	16	16
8	NE8	No.Trend	No.Trend	No.Trend	No.Trend	No.Trend	No.Trend	No.Trend	Upward	No.Trend	No.Trend	No.Trend	No.Trend	16	16	16	16	16	16	16	16	16	16	16	16
9	NE9	No.Trend	No.Trend	No.Trend	No.Trend	No.Trend	No.Trend	No.Trend	Upward	No.Trend	No.Trend	No.Trend	No.Trend	16	16	16	16	16	16	16	16	16	16	16	16
10	NE10	No.Trend	No.Trend	No.Trend	No.Trend	No.Trend	No.Trend	No.Trend	Upward	No.Trend	No.Trend	No.Trend	No.Trend	16	16	16	16	16	16	16	16	16	16	16	16
11	NE11	No.Trend	No.Trend	No.Trend	No.Trend	No.Trend	No.Trend	No.Trend	Upward	No.Trend	No.Trend	No.Trend	No.Trend	15	15	16	16	16	16	16	16	15	15	15	15
12	SM1	No.Trend	No.Trend	No.Trend	No.Trend	No.Trend	No.Trend	No.Trend	No.Trend	No.Trend	No.Trend	No.Trend	No.Trend	16	16	16	16	16	16	16	16	16	16	16	16
13	SM2	No.Trend	No.Trend	No.Trend	No.Trend	No.Trend	No.Trend	No.Trend	Upward	No.Trend	No.Trend	No.Trend	No.Trend	16	16	16	16	16	16	16	16	16	16	16	16
14	SM3	No.Trend	No.Trend	No.Trend	No.Trend	No.Trend	No.Trend	No.Trend	No.Trend	No.Trend	No.Trend	No.Trend	No.Trend	16	16	16	16	16	16	16	16	16	16	16	16
15	SM4	No.Trend	No.Trend	No.Trend	No.Trend	No.Trend	No.Trend	No.Trend	No.Trend	No.Trend	No.Trend	No.Trend	No.Trend	16	16	16	16	16	16	16	16	16	16	16	16
16	SM5	No.Trend	No.Trend	No.Trend	No.Trend	No.Trend	No.Trend	No.Trend	No.Trend	No.Trend	No.Trend	No.Trend	No.Trend	16	16	16	16	16	16	16	16	16	16	16	16
17	SM6	No.Trend	No.Trend	No.Trend	No.Trend	No.Trend	No.Trend	No.Trend	No.Trend	No.Trend	No.Trend	No.Trend	No.Trend	16	16	16	16	16	16	16	16	16	16	16	16
18	SM7	No.Trend	No.Trend	No.Trend	No.Trend	No.Trend	No.Trend	No.Trend	Upward	No.Trend	Upward	No.Trend	Downward	16	16	16	16	16	16	16	16	16	16	16	16
19	SM8	No.Trend	No.Trend	No.Trend	No.Trend	No.Trend	No.Trend	No.Trend	No.Trend	No.Trend	No.Trend	No.Trend	No.Trend	16	16	16	16	16	16	16	16	16	16	16	16
20	SM9	No.Trend	No.Trend	No.Trend	No.Trend	No.Trend	No.Trend	No.Trend	No.Trend	No.Trend	No.Trend	No.Trend	No.Trend	16	16	16	16	16	16	16	16	16	16	16	16
21	SM10	No.Trend	No.Trend	No.Trend	No.Trend	No.Trend	No.Trend	No.Trend	No.Trend	No.Trend	No.Trend	No.Trend	No.Trend	16	16	16	16	16	16	16	16	16	16	16	16
22	WF1	No.Trend	No.Trend	No.Trend	No.Trend	No.Trend	No.Trend	No.Trend	No.Trend	No.Trend	No.Trend	No.Trend	No.Trend	16	16	16	16	16	16	16	16	16	16	16	16
23	WF2	No.Trend	No.Trend	No.Trend	No.Trend	No.Trend	No.Trend	No.Trend	No.Trend	No.Trend	No.Trend	No.Trend	No.Trend	16	16	16	16	16	16	16	16	16	16	16	16
24	WF3	No.Trend	No.Trend	No.Trend	No.Trend	No.Trend	No.Trend	No.Trend	Upward	No.Trend	No.Trend	No.Trend	No.Trend	16	16	16	16	16	16	16	16	16	16	16	16
25	WF4	No.Trend	No.Trend	No.Trend	No.Trend	No.Trend	No.Trend	No.Trend	Upward	No.Trend	No.Trend	No.Trend	No.Trend	16	16	16	16	16	16	16	16	16	16	16	16
26	WF5	No.Trend	No.Trend	No.Trend	No.Trend	No.Trend	No.Trend	No.Trend	Upward	No.Trend	No.Trend	No.Trend	No.Trend	16	16	16	16	16	16	16	16	16	16	16	16
27	WF6	No.Trend	No.Trend	No.Trend	No.Trend	No.Trend	No.Trend	No.Trend	Upward	No.Trend	No.Trend	No.Trend	No.Trend	16	16	16	16	16	16	16	16	16	16	16	16
28	WF7	No.Trend	No.Trend	No.Trend	No.Trend	No.Trend	No.Trend	No.Trend	Upward	No.Trend	No.Trend	No.Trend	No.Trend	16	16	16	16	16	16	16	16	16	16	16	16
29	WF8	No.Trend	No.Trend	No.Trend	No.Trend	No.Trend	No.Trend	No.Trend	Upward	No.Trend	No.Trend	No.Trend	No.Trend	15	15	16	16	16	16	16	16	15	15	15	15
30	BM1	No.Trend	No.Trend	No.Trend	No.Trend	No.Trend	No.Trend	No.Trend	Upward	No.Trend	No.Trend	No.Trend	No.Trend	16	16	16	16	16	16	16	16	16	16	16	16
31	BM2	No.Trend	No.Trend	No.Trend	No.Trend	No.Trend	No.Trend	No.Trend	Upward	No.Trend	Upward	No.Trend	Downward	16	16	16	16	16	16	16	16	16	16	16	16
32	BM3	No.Trend	No.Trend	No.Trend	No.Trend	No.Trend	No.Trend	No.Trend	Upward	No.Trend	No.Trend	No.Trend	No.Trend	16	16	16	16	16	16	16	16	16	16	16	16
33	BM4	No.Trend	No.Trend	No.Trend	No.Trend	No.Trend	No.Trend	No.Trend	Upward	No.Trend	No.Trend	No.Trend	No.Trend	16	16	16	16	16	16	16	16	16	16	16	16
34	BM5	No.Trend	No.Trend	No.Trend	No.Trend	No.Trend	No.Trend	No.Trend	Upward	No.Trend	No.Trend	No.Trend	No.Trend	16	16	16	16	16	16	16	16	16	16	16	16
35	BM6	No.Trend	No.Trend	Upward	Upward	No.Trend	No.Trend	No.Trend	Upward	No.Trend	No.Trend	No.Trend	No.Trend	16	16	16	16	16	16	16	16	16	16	16	16
36	BM7	Upward	Upward	No.Trend	No.Trend	No.Trend	No.Trend	No.Trend	Upward	Upward	No.Trend	No.Trend	No.Trend	16	16	16	16	16	16	16	16	16	16	16	16
37	BM8	No.Trend	No.Trend	No.Trend	No.Trend	No.Trend	No.Trend	No.Trend	Upward	No.Trend	No.Trend	No.Trend	No.Trend	16	16	16	16	16	16	16	16	16	16	16	16
38	BM9	No.Trend	No.Trend	No.Trend	No.Trend	No.Trend	No.Trend	No.Trend	Upward	No.Trend	No.Trend	No.Trend	No.Trend	16	16	16	16	16	16	16	16	16	16	16	16
39	BM10	No.Trend	No.Trend	No.Trend	No.Trend	No.Trend	No.Trend	No.Trend	Upward	No.Trend	No.Trend	No.Trend	No.Trend	16	16	16	16	16	16	16	16	16	16	16	16
40	BM11	No.Trend	No.Trend	No.Trend	No.Trend	No.Trend	No.Trend	No.Trend	Upward	No.Trend	No.Trend	No.Trend	No.Trend	16	16	16	16	16	16	16	16	16	16	16	16
41	CM1	No.Trend	No.Trend	Upward	Upward	No.Trend	No.Trend	No.Trend	Upward	No.Trend	No.Trend	No.Trend	No.Trend	15	15	16	16	16	16	16	16	15	15	15	15
42	CM2	No.Trend	No.Trend	No.Trend	No.Trend	No.Trend	No.Trend	No.Trend	No.Trend	No.Trend	No.Trend	No.Trend	No.Trend	15	15	16	16	16	16	16	16	15	15	15	15
43	CM3	No.Trend	No.Trend	Upward	Upward	No.Trend	No.Trend	No.Trend	Upward	No.Trend	No.Trend	No.Trend	No.Trend	15	15	16	16	16	16	16	16	15	15	15	15
44	CM4	No.Trend	No.Trend	No.Trend	No.Trend	No.Trend	No.Trend	No.Trend	Upward	No.Trend	No.Trend	No.Trend	No.Trend	15	15	16	16	16	16	16	16	15	15	15	15
45	CM5	No.Trend	No.Trend	No.Trend	No.Trend	No.Trend	No.Trend	No.Trend	No.Trend	No.Trend	No.Trend	No.Trend	No.Trend	15	15	16	16	16	16	16	16	15	15	15	15
46	S1	No.Trend	Upward	No.Trend	No.Trend	No.Trend	No.Trend	No.Trend	Upward	No.Trend	No.Trend	No.Trend	No.Trend	16	16	16	16	16	16	16	16	16	16	16	16
47	S2	No.Trend	No.Trend	No.Trend	No.Trend	No.Trend	Downward	No.Trend	No.Trend	No.Trend	No.Trend	No.Trend	No.Trend	15	15	16	16	16	16	16	16	15	15	15	15
48	S3	No.Trend	No.Trend	Upward	No.Trend	No.Trend	No.Trend	No.Trend	No.Trend	No.Trend	No.Trend	No.Trend	No.Trend	16	16	16	16	16	16	16	16	16	16	16	16
49	S4	No.Trend	No.Trend	Upward	No.Trend	No.Trend	No.Trend	No.Trend	No.Trend	No.Trend	No.Trend	No.Trend	No.Trend	16	16	16	16	16	16	16	16	16	16	16	16
50	S5	No.Trend	Upward	No.Trend	No.Trend	No.Trend	Downward	No.Trend	No.Trend	No.Trend	No.Trend	Downward	No.Trend	15	15	16	16	16	16	16	16	15	15	15	15

## 2 Experimental design, materials and methods

### 2.1 Water sampling and analysis

Acid-sensitive lakes were selected by the Regional Aquatics Monitoring Group from an initial regional survey of 449 lakes to be representative of lake and watershed characteristics and chemistry across six sub-regions within the study area [27]. Lakes were generally situated in remote locations accessible only by fixed-wing aircraft or helicopter. Water samples for analysis of the stable isotopes of water were collected for the purpose of establishing site-specific and year-specific water yield to lakes using an isotope balance method [12]. This, combined with concurrent geochemical sampling for base cations, was designed to enable estimation of critical loads of acidity to the lakes using a simple steady-state water chemistry model [28]. Critical loads of acidity is a measure of the buffering capacity of the lake-watershed system to potential acidifying emissions. In the case of the RAMP lakes network, assessment of potential impacts from emissions from oil sands operations on local watersheds and lakes was the primary objective of annual time-series monitoring. One complicating factor realized in the second decade of monitoring was the significant impact of permafrost thaw on runoff to many lakes in the Birch Mountains, Caribou Mountains and Northeast of Fort McMurray [see 1,4], which may significantly and differentially affect the long-term representativeness of the site-specific critical load of acidity calculations.

For deeper lakes (>3 m), lake water samples were collected near the centre of the major basin at a single deep-water site using weighted Tygon tubing and a one-way valve. This approach was used to provide vertically-integrated samples representative of the euphotic zone (defined as twice the Secchi disk depth). For shallow lakes (<3 m deep), composite samples were created from five to ten 1 L grab samples collected at 0.5 m depth along an upwind to downwind transect. Samples taken from a given lake were then combined to form a single composite sample. Euphotic zone samples from deep lakes, and composite samples from shallow lakes were then split according to requirements for specific analyses including an unfiltered, 30-mL sample in a high-density polyethylene bottle for stable isotope analysis, as well as various bottles for geochemical analyses. All bottles were subsequently refrigerated and returned to various labs for analysis (Colin Cooke, Alberta Environment and Parks, pers. Comm.)

### 2.2 Water balance data

Lake area, watershed area and NARR monthly climatology parameters (precipitation, temperature, relative humidity, evaporation and precipitation) were used in combination with isotopic data to estimate annual lake water balance by an established isotopic method [12]. Input to lakes was estimated based on amount-weighted isotopic composition of precipitation. Isotopic composition of atmospheric moisture was defined using the partial equilibrium approach [4], which involved fitting predicted oxygen-18 and deuterium enrichment to match the slope of the local evaporation line [1]; see also [29].
